# Sex differences in mouse infralimbic cortex projections to the nucleus accumbens shell

**DOI:** 10.1186/s13293-023-00570-3

**Published:** 2023-12-11

**Authors:** Caroline S. Johnson, Andrew D. Chapp, Erin B. Lind, Mark J. Thomas, Paul G. Mermelstein

**Affiliations:** 1 Department of Neuroscience, School of Medicine, University of Minnesota, 4-140 Jackson Hall, 321 Church St SE, Minneapolis, MN 55455 USA; 2 Medical Discovery Team on Addiction, University of Minnesota, 3-432 McGuire Translational Research Facility, 2001 6th St SE, Minneapolis, MN 55455 USA

**Keywords:** Sex differences, Glutamate, Infralimbic cortex, Nucleus accumbens shell, Motivation, Reward, Intracranial self-stimulation, Optogenetics, Intrinsic excitability, Synaptic strength

## Abstract

**Background:**

The nucleus accumbens (NAc) is an important region in motivation and reward. Glutamatergic inputs from the infralimbic cortex (ILC) to the shell region of the NAc (NAcSh) have been implicated in driving the motivation to seek reward through repeated action-based behavior. While this has primarily been studied in males, observed sex differences in motivational circuitry and behavior suggest that females may be more sensitive to rewarding stimuli. These differences have been implicated for the observed vulnerability in women to substance use disorders.

**Methods:**

We used an optogenetic self-stimulation task in addition to ex vivo electrophysiological recordings of NAcSh neurons in mouse brain slices to investigate potential sex differences in ILC-NAcSh circuitry in reward-seeking behavior. Glutamatergic neurons in the ILC were infected with an AAV delivering DNA encoding for channelrhodopsin. Entering the designated active corner of an open field arena resulted in photostimulation of the ILC terminals in the NAcSh. Self-stimulation occurred during two consecutive days of testing over three consecutive weeks: first for 10 Hz, then 20 Hz, then 30 Hz. Whole-cell recordings of medium spiny neurons in the NAcSh assessed both optogenetically evoked local field potentials and intrinsic excitability.

**Results:**

Although both sexes learned to seek the active zone, within the first day, females entered the zone more than males, resulting in a greater amount of photostimulation. Increasing the frequency of optogenetic stimulation amplified female reward-seeking behavior. Males were less sensitive to ILC stimulation, with higher frequencies and repeated days required to increase male reward-seeking behavior. Unexpectedly, ex vivo optogenetic local field potentials in the NAcSh were greater in slices from male animals. In contrast, female medium-spiny neurons (MSNs) displayed significantly greater intrinsic neuronal excitability.

**Conclusions:**

Taken together, these data indicate that there are sex differences in the motivated behavior driven by glutamate within the ILC-NAcSh circuit. Though glutamatergic signaling was greater in males, heightened intrinsic excitability in females appears to drive this sex difference.

**Supplementary Information:**

The online version contains supplementary material available at 10.1186/s13293-023-00570-3.

## Highlights

- There are sex differences in the behavioral output of optogenetic intracranial self-stimulation of glutamatergic projections from the infralimbic cortex to the shell of the nucleus accumbens (NAcSh). Females exhibit greater sensitivity to the stimulation of this circuit than do males.
- Ex vivo optogenetically evoked local field potentials in MSNs in the NAcSh also show intrinsic sex differences, with males exhibiting significantly greater responses than females; counterintuitive to the behavioral data.
- Intrinsic neuronal excitability of NAcSh MSNs is greater in females than in males, which may account for the observed behavioral differences.

**Supplementary Information:**

The online version contains supplementary material available at 10.1186/s13293-023-00570-3.

## Abstract (plain language summary)

The shell region of the nucleus accumbens (NAcSh) is involved in motivation and reward. It receives excitatory glutamatergic inputs from multiple brain regions. One specific region is the infralimbic cortex (ILC), which when activated, influences reward-seeking behavior. While previous research has focused on males, there are inherent sex differences in reward circuitry and reward-seeking behavior. Using an optogenetic self-stimulation task, in addition to ex vivo electrophysiological recordings, we found inherent sex differences in the ILC-NAcSh circuit in behavioral output, synaptic strength, and intrinsic neurophysiology. Female mice showed more robust reward-seeking behavior. Increasing the frequency of stimulation intensified this behavior in females, while males required higher frequencies and repeated testing days to increase their reward-seeking behavior. Surprisingly, optogenetically stimulating the ILC terminals in the NAcSh in brain slices resulted in stronger responses in males. More consistent with the behavioral data, female MSNs displayed higher intrinsic excitability. Our results suggest that there are sex differences in motivated behavior, driven by glutamatergic signaling in the ILC-NAc circuit. Despite stronger ILC-based glutamatergic signaling in males, heightened intrinsic excitability of MSNs in females seems to be the driving force behind this sex difference in reward-seeking behavior. These findings contribute to our understanding of the neural mechanisms behind sex-based differences in motivation and their potential implications for substance use disorders.

**Supplementary Information:**

The online version contains supplementary material available at 10.1186/s13293-023-00570-3.

## Background

The nucleus accumbens (NAc) is a critical region in motivation for reward and incentive salience [1]. Part of the mesolimbic reward circuit, the NAc receives inputs from midbrain, limbic, hypothalamic, and cortical regions that are integrated to drive goal-directed behavioral responses to rewarding stimuli [2–4]. The NAc can be divided into the core (NAcC) and shell (NAcSh) regions. The shell encodes reward that translates motivation into behavior to seek that reward [5, 6]. At the most basic level, motivated behaviors are those in pursuit of fundamental needs, allowing for the continued survival of the individual and/or the species [7]. The underlying circuitry modulates all motivated behaviors, both adaptive (e.g., seeking food, copulation) and maladaptive (e.g., seeking drugs of abuse). Specifically, the NAc is sensitive to drugs of abuse, producing aberrations in synaptic activity that have been implicated in the etiology of substance use disorders [8].

While the dopaminergic input to the NAc via innervation from the ventral tegmental area (VTA) of the midbrain has been extensively studied [9], the region receives significant glutamatergic input as well [10, 11], including dense excitatory projections from the infralimbic cortex (ILC) [12]. The ILC, a subdivision of the prefrontal cortex, has been implicated in complex and often contradictory roles, including in the development of habitual behaviors [12–14] and in the acquisition of extinction learning [15–17]. Stimulation of the glutamatergic ILC-NAcSh projections has been shown to be involved in reinforcing reward-oriented action-based behaviors [18], while changes in synaptic efficacy in the ILC-NAcSh circuit have been observed in rodent models of relapse [19, 20]. Furthermore, optogenetic stimulation of the ILC-NAcSh circuit has been shown to be involved in the reinstatement of cocaine-induced conditioned place preference, while inhibiting this pathway blocked reinstatement [21].

Furthermore, significant sex differences in the motivation and reward system have been found [22]. In humans, women tend to reach compulsive drug use at a faster rate than men [23], and report more occurrences of spontaneous relapse [24–26]. In rodent models of psychostimulant self-administration, females learn to self-administer faster, work harder for drug, exhibit more patterned locomotion, and are more sensitive to its rewarding properties [27–29]. Additionally, both stress- and cue-induced reinstatement of drug-seeking behaviors is greater in females than in males [30, 31].

Part of the mechanism that underlies these sex differences seems to include glutamatergic signaling. Sex differences in glutamatergic signaling have been found in both clinical and preclinical populations [32]. Rodent studies have shown sex differences in glutamate concentrations in multiple brain regions, with additional differences across the estrous cycle [33]. Within the medial prefrontal cortex (mPFC), females also exhibit a higher amplitude and frequency of spontaneous excitatory postsynaptic currents, as well as greater inward rectification [34]. Like the ILC, the mPFC is a glutamatergic region that sends projections to the NAc, including both the core and shell regions [35–37], and sex differences in glutamate plasticity have been demonstrated within the NAc following cocaine administration [38].

Female medium-spiny neurons (MSNs) of the NAcC display greater spine density than male MSNs [39]. Sex differences in spine density in the NAc are found in humans as well [40]. Estradiol and estrogen receptor signaling modulates striatal glutamatergic activity [39, 41–43], yet as whole, the striatum (including the NAc) is largely devoid of nuclear estrogen receptors (ERs) [44, 45]. This suggests that the actions of estradiol occur through membrane-localized ERs (mERs). Indeed, glutamate signaling in the female NAc occurs through the functional coupling of mERs and metabotropic glutamate receptors (mGluRs) [46].

In the core of the NAc, estradiol decreases dendritic spine density through activation of mGluR5 signaling [47, 48]. The effects of estradiol in the NAcSh have been more varied [47], with some studies having found an increase in spine density through estradiol transactivation of mGluR1a [48]. In fact, changes in functional glutamatergic input have been shown to occur throughout the estrous cycle, coincident with changes in dendritic spine plasticity in MSNs in both the NAcC and NAcSh [49]. Estradiol regulates many of these functional changes through ERα [50, 51]. When compared with males, female MSNs display higher mEPSC frequency prepubertally, indicating an organizational nature of these sex differences as well [52]. Furthermore, in the adult NAcC, females exhibit higher AMPA/NMDA ratios [53]. Additionally, females have a larger readily releasable pool of glutamate, but display a lower release probability [53].

While the glutamatergic ILC-NAcSh pathway exhibits sex differences [54–56], the full picture of how this circuitry differentially influences motivated behavior remains unclear. In the present study we used an optogenetic intracranial self-stimulation task [18], along with electrophysiological recordings, to investigate sex differences in ILC-NAcSh circuitry and parse out differing behavioral strategies in reward-seeking behavior. Our results demonstrate sex differences in motivated behavior, synaptic, and intrinsic neuronal excitability within the circuit. Females exhibit more robust motivated behavior upon activation of ILC terminals in the NAcSh, correlated with heightened MSN intrinsic excitability. In contrast, males display stronger ILC to NAcSh synaptic strength.

## Methods

### Animals

**Fig. 1:**
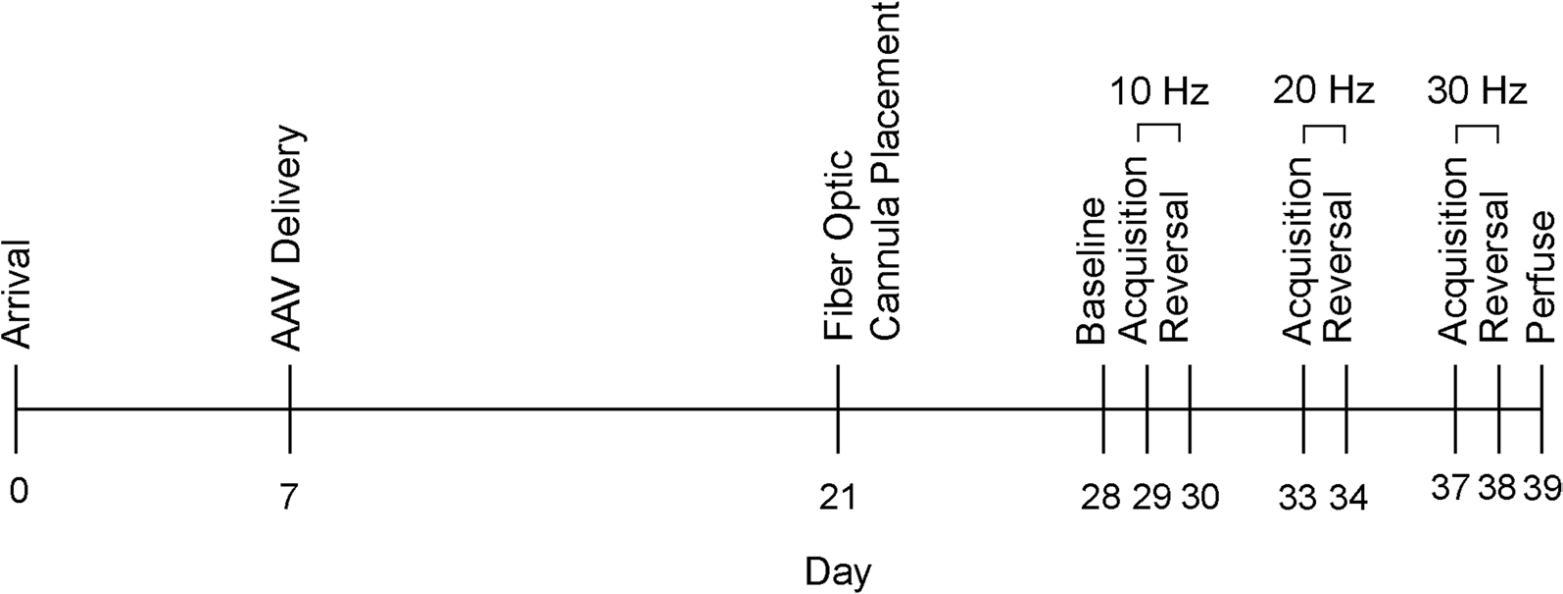
Timeline of the behavioral experiment. Seven days after arrival, mice were received viral delivery of channelrhodopsin. The fiber optic cannulae were placed on day 21. Behavioral testing began on approximately day 28 with the baseline test, followed by 10 Hz acquisition then reversal trials. 20 Hz acquisition and reversal trials, then 30 Hz acquisition and reversal trials followed spaced approximately one week apart, and for females, in accordance with the appropriate phases of the estrous cycle. Mice were perfused following the final behavioral trial

A total of 24 adult (≥ P60) female and 20 adult male C57BL/6J mice (Jackson Laboratories, #000664) were used. Of these mice, 18 females and 14 males underwent behavioral testing. Mice used for electrophysiological studies were naïve to behavioral testing. For optogenetic electrophysiology, three females and three males were used, and to measure neuronal excitability, a total of 10 cells were used from females (*n* = 3 mice) and 10 cells from males (*n *= 3 mice). Mice were housed in a temperature- and humidity-controlled vivarium under a reverse 12:12 light:dark cycle. All measurements were made during the dark phase of the cycle. Mice were group-housed 2–4 to a cage and allowed to acclimate to the facility for 7 days before beginning any procedure. All experimental procedures were approved by the University of Minnesota Institutional Animal Care and Use Committee, and followed guidelines set by the American Association for the Accreditation of Laboratory Animal Care. The experimental timeline is shown in Fig. 1.

### Surgery

Mice undergoing behavioral testing or ex vivo optogentic stimulation were anaesthetized using isoflurane and transfected with pAAV-CaMKIIa-ChR2(H134R)-EYFP (Addgene #26,969-AAV2). The AAV promoter CaMKIIa exhibits selectivity for excitatory glutamatergic neurons [57–59]. AAVs were delivered bilaterally into the ILC (from bregma: AP: + 1.80 ML: ± 0.40 DV: − 3.10) with a 10 μl Hamilton syringe equipped with a removable 34-gauge needle (Hamilton Company, #207,434–10), via a World Precision Instruments Microinjection Syringe Pump controlled by the SMARTouch Controller (World Precision Instruments UMP 3 T-1). A total volume of 500 nL was delivered at a rate of 100 nL/min. Two weeks later, custom-made borosilicate ferrule fibers (200 μm fiber diameter, 0.66 NA borosilicate fiber, 1.25 mm zirconia receptacle, Doric Lenses) were implanted bilaterally above the NAcSh (from bregma: AP: + 1.50 ML: ± 1.63 DV: − 4.10) and fixed to the skull using dental cement (Metabond, Parkell). Mice used for optogenetic local field potentials were transfected with the AAV as described above but did not receive fiber optic implants. Mice used for the whole-cell current clamp experiment underwent no surgical manipulations. Animals were allowed to recover from surgery for 7 days before behavioral testing.

### Estrous cycle tracking

Vaginal lavage was used to track estrous cycle in the female mice. Mice showed regular 4–5 day cycles. Behavior testing was run in accordance with the estrous cycle; among females, Group 1 underwent baseline testing during diestrus, acquisition testing during proestrus, and reversal testing in estrous, and Group 2 underwent baseline testing in proestrus, acquisition in estrous, and reversal in metestrus (Table 1). Male mice were matched and tested on the same schedule as females. As no behavioral differences were found across the estrous cycle (see Results), electrophysiological recordings were done during estrous as this cycle spanned both behavioral groups.

**Table 1:** Behavioral groups

Group	Sex	Phase at baseline	Phase at acquisition	Phase at reversal	*n*
Group 1	Female	Diestrus	Proestrus	Estrus	10
Group 2	Female	Proestrus	Estrus	Metestrus	8
Group 3	Male	–	–	–	14

It presents the group parameters and the number of subjects in each behavioral group

For the female groups (1 and 2), the stage of estrous cycle in which each phase of the trial occurred is presented

### Behavior

Experimental details regarding intracranial self-stimulation were similar to those previously described [18] with two exceptions. In contrast to the prior study, mice were tested under red light within 2 h of when colony lights were turned off. Frequency response assessment was also done using a within-subject approach, whereas prior assessments were done using a between-subject approach. Control data indicated no repeated measures effect, in addition to no ordering effect, hence the use of a within-subject design. Specifically, we compared the 30-Hz stimulation data presented here with male and female mice that received only 30 Hz stimulation, with no differences found in the number of entries into the active zone, the time spent in the active zone, the number of stimulations received, the duration of optogenetic stimulation, distance traveled, or speed (data not shown). An open field arena (20 in × 20 in × 8 in) with isolated corner zones (6 in × 6 in) containing different context cues (triangles, dots, horizontal lines, or vertical lines) was used, placed in the same orientation for every test. The implanted optogenetic fiber ferrules were connected to patch cables above the arena and mice were allowed free movement within the arena. PlexBright LEDs (Plexon) were used to deliver blue light (465 nm, 5 ms pulse width, 10–15 mW, 4–6 mW/mm^2^) bilaterally through the patch cables (200 μm, 0.66 NA, Plexon), which were connected to the implanted fiber ferrules via zirconia sleeves (1.25 mm OD, Plexon). Over the course of the experiment, mice were exposed to 10 Hz, 20 Hz, and 30 Hz stimulation parameters sequentially, resulting in 7 total days of behavior testing. ANY-maze software (Stoelting Co.) was used to control optogenetic stimulation and record behavioral video data from cameras located directly above each arena. During each trial (except for Baseline), a single corner was designated as the active zone, and entry into this corner triggered optogenetic stimulation. The remaining three corners did not elicit optogenetic stimulation, but rather “mock” stimulation in which the software recorded all the same parameters as the active zone. Upon entering the designated active zone, optogenetic stimulation was triggered. Stimulation was provided for a total of 5 s, then shut off for 15 s. If the mouse stayed in the active zone during this 15 s timeout period, the cycle restarted, replaying the 5 s of stimulation and 15 s of no stimulation. This cycle was possible for the entire 30-min trial. However, the 15 s timeout could be bypassed by exiting the zone and re-entering, thereby re-starting the stimulation cycle.

### Baseline

Mice were initially habituated to the arena during a 30-min baseline trial. During this time the patch cables were connected to the implanted fiber ferrules, but no zone triggered an optogenetic response. The time each mouse spent in each of the four quadrants was recorded. Acquisition and reversal zones were counterbalanced using quadrants that were not the most- or least-preferred as determined by baseline testing.

### Acquisition

Acquisition trials (i.e., day 1 of stimulation at a particular frequency) lasted 30 min and were run 3 times across the experiment. Each mouse was assigned an active quadrant, and entry into the zone would elicit the stimulation parameters outlined above. As mentioned, each acquisition trial was in either proestrus (Group 1) or estrus (Group 2). Males were run on the same schedule as females. The first acquisition trial utilized 10 Hz stimulation, the second acquisition trial was run with 20 Hz stimulation, and the final acquisition trial was run with 30 Hz stimulation.

### Reversal

Reversal trials occurred the day after an acquisition trial (i.e., day 2 of stimulation at the same frequency). For all females involved, reversal trials occurred during either estrus (Group 1) or metestrus (Group 2). Again, male mice were matched and run on the same schedule as females. The active zone was switched to a previously inactive quadrant, and entry into this new stimulation zone elicited the same programmed parameters as were utilized in acquisition. As with acquisition trials, the first reversal trial delivered 10-Hz stimulation, the second trial delivered 20-Hz stimulation, and the third trial delivered 30-Hz stimulation. Following the final 30-Hz reversal trial, mice were perfused and brains underwent processing for sectioning (see below).

### Video analysis

ANY-maze software recorded multiple analysis parameters, including the number of entries into each quadrant, the total time spent in each quadrant, the number of optogenetic stimulations received in each quadrant (actual or mock), as well as the total time optogenetic stimulation was received (actual or mock). Mean distance traveled and mean travel speed during each trial was assessed to analyze activity levels. For all acquisition trials, the average number of entries made into any of the inactive zones was determined and subtracted from the number of entries made into the active zone to determine if mice were indeed entering the active zone preferentially or if they were seeking all zones equally. This calculation was repeated for time spent in the zones. Though “mock stimulations” were collected by the ANY-maze software, we did not subtract the number of stimulations received in the acquisition zone from the average of mock stimulations received in inactive zones, nor did we subtract the average amount of time the stimulation was received from the average of the amount of time these mock stimulations were received. This measurement had no practical meaning for the mice, and as such it was sufficient to measure only the actual values to determine if the mice were seeking the stimulation in the acquisition zone.

During the reversal trials, the number of entries made into the acquisition day’s active zone (“Zone 1”) was subtracted from the number of entries made into the new active zone (“Zone 2”), to determine if mice sought the new active zone, or if they continued to seek the previous active zone.

### Perfusion and imaging

**Fig. 2:**
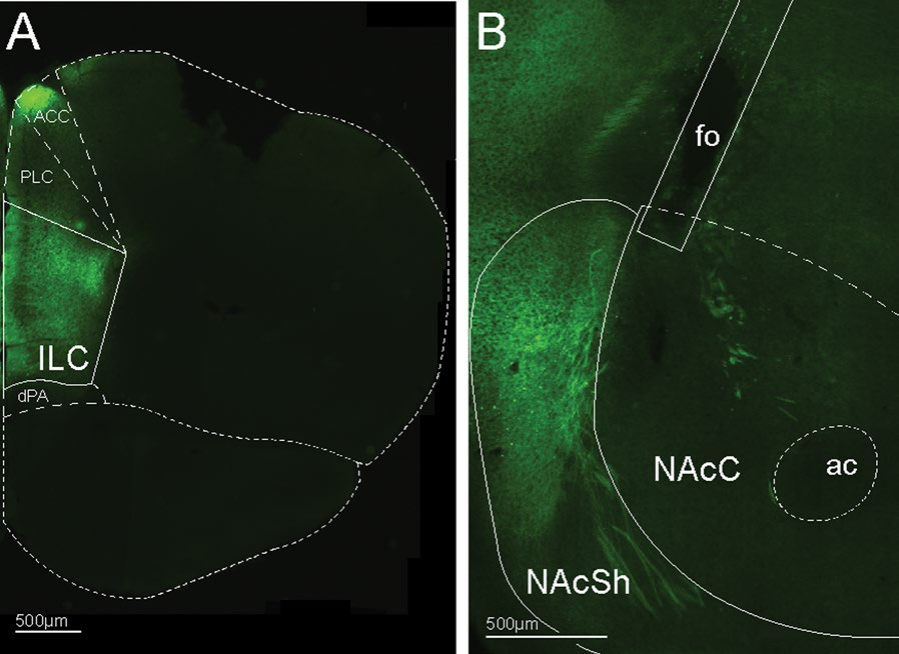
AAV and fiber optic placement. **A** Representative image of targeted AAV injection, with cell bodies in the infralimbic cortex (ILC) expressing eYFP-ChR2. **B** Representative image of fiber optic cannula placement above the nucleus accumbens shell (NAcSh). Projections from the ILC are in green. Fiber optic placement (fo) indicated by the solid lines. ac, anterior commissure; ACC, anterior cingulate cortex; dPA, dorsal peduncular area; fo, fiber optic cannula; ILC, infralimbic cortex; NAcC, nucleus accumbens core; NAcSh, nucleus accumbens shell; PLC, prelimbic cortex. Scale bars indicate 500 μm

Following the final 30-Hz reversal behavioral test, mice were deeply anaesthetized with isoflurane and transcardially exsanguinated with cold 1X PBS (pH 7.5), followed by perfusion with cold 4% paraformaldehyde (PFA; pH 7.5) in 1X PBS. Brains were removed and post-fixed in the same PFA solution for 24 h at 4 °C, before being switched to a 30% (weight/volume) sucrose solution in 1X PBS for 24 h, again at 4 °C. Brains were then flash-frozen using hexanes (ThermoFisher Scientific, Catalog #: 423,765,000) cooled on dry ice [60, 61]. Brains were next sectioned (25 μm thickness) on a cryostat (Leica, CM1800) and mounted onto Kemtech White Glass Slides (Kemtech, 0313–7121) to determine AAV and fiber optic placement. Brains were imaged using an epifluorescent microscope (Leica, DM4000B) to determine AAV and fiber optic cannula placement. Only mice with correctly placed AAVs and fiber optic cannulae were included in statistical analyses (Fig. 2).

### Electrophysiological recordings

To compare MSN neuronal excitability between female and male mice, we prepared brain slices for electrophysiological recordings. These recordings were made from the medial NAc shell, the same region that was targeted in our in vivo experiments. Mice were anesthetized with isoflurane (3% in O_2_) and decapitated during the dark phase of the light cycle. Brains were rapidly removed and chilled in ice cold cutting solution, containing (in mM): 228 sucrose, 2.5 KCl, 7 MgSO_4_, 1.0 NaH_2_PO_4_, 26 NaHCO_3_, 0.5 CaCl_2_, 11 d-glucose, pH 7.3–7.4, continuously gassed with 95:5 O_2_:CO_2_ to maintain pH and pO_2_. A brain block including the NAcSh region was cut and affixed to a vibrating microtome (Leica VT 1000S; Leica, Nussloch, Germany). Sagittal sections of 240 µm thickness were cut, and the slices were allowed to recover for 1 h in standard ACSF continuously gassed with 95:5 O_2_:CO_2_ containing (in mM): 119 NaCl, 2.5 KCl, 1.3 MgSO_4_, 1.0 NaH_2_PO_4_, 26.2 NaHCO_3_, 2.5 CaCl_2_, 11 d-glucose, and 1.0 ascorbic acid (osmolarity: 295–302 mosmol L^−1^; pH 7.3–7.4). Following recovery, slices were transferred to a glass-bottomed recording chamber circulated at a rate of 2 ml min^−1^ with standard ACSF, continuously gassed with 95:5 O_2_:CO_2_. Slices were viewed through an upright microscope (Olympus) equipped with DIC optics, an infrared (IR) filter and an IR-sensitive video camera (DAGE-MTI).

Patch electrodes (Flaming/Brown P-97, Sutter Instrument, Novato, CA) were pulled from borosilicate glass capillaries with a tip resistance of 5–10 MΩ. Electrodes were filled with a solution containing (in mM) 135 K-gluconate, 10 HEPES, 0.1 EGTA, 1.0 MgCl_2_, 1.0 NaCl, 2.0 Na_2_ATP, and 0.5 Na_2_GTP (osmolarity: 280–285 mosmol L^−1^; pH 7.3) [62–65]. MSNs were identified under IR-DIC based on their morphology and hyperpolarizing membrane potential (-70 to -80 mV). MSNs were voltage clamped at -80 mV using a Multiclamp 700B amplifier (Molecular Devices), and the currents were filtered at 2 kHz and digitized at 10 kHz. Holding potentials were not corrected for the liquid junction potential. Once a GΩ seal was obtained, slight suction was applied to break into whole-cell configuration and the cell was allowed to stabilize. Stability was determined by monitoring capacitance, membrane resistance, access resistance (from membrane test window) and resting membrane potential (V_m_) [65, 66]. Records were not corrected for a liquid junction potential of -15 mV. Cells that met the following criteria were included in the analysis: action potential amplitude ≥ 50 mV from threshold to peak, resting *V*_m_ negative to − 64 mV, and < 20% change in series resistance during the recording.

To measure NAcSh MSN neuronal excitability, V_m_ was adjusted to -80 mV by continuous negative current injection. A series of square-wave current injections were delivered in steps of + 20 pA, each for a duration of 800 ms (ms). To determine the action potential voltage threshold (Vt), rheobase, and time to action potential (AP), ramp current injections (0.437 pA/ms, 800 ms) were made from a holding potential of -80 mV. To determine input resistance, a square-wave current injection (− 20 pA/800 ms) was made, and input resistance calculated using ohm’s law. Square-wave, and ramp current injections were made in the same neurons.

In a separate group of animals which had AAV-ChR2 in the ILC, we assessed ILC-NAcSh glutamatergic synaptic strength using optogenetic evoked local field potentials (oLFPs). Brain slices were transferred to a recording chamber in standard ACSF with picrotoxin (100 µM) to block GABA currents, continuously gassed with 95:5 O_2_:CO_2_ containing (in mM): 119 NaCl, 2.5 KCl, 1.3 MgSO_4_, 1.0 NaH_2_PO_4_, 26.2 NaHCO_3_, 2.5 CaCl_2_, 11 d-glucose, and 1.0 ascorbic acid (osmolarity: 295–302 mosmol L^−1^; pH 7.3–7.4). The pipette (tip resistance ~ 0.5–1.0 MΩ) was also filled with standard ACSF. To stimulate oLFPs, a SOLA SE light engine (3.5–4 watts power, Beaverton, OR), equipped with the appropriate fluorescent filter was triggered via Clampex (Molecular Devices) every 4 s through the microscope objective (10 × magnification power) with a total sweep duration of 30 s. Light pulse time was increased from 1.0–4.0 ms. Recordings were low-pass filtered (0.3 kHz), high-pass filtered (0.1 Hz) and Bessel filtered (30 kHz). Traces were analyzed offline using Clampfit software (Molecular Devices). Each oLFP data point was from a single slice and typically one animal produced 2–3 useable slices. Note that neuronal excitability and oLFP recordings were collected in separate animals. Passive membrane properties of MSNs in NAcSh are provided in Table 2.

**Table 2:** Passive membrane properties

	Capacitance (pF)	Resting membrane potential (mv)	Membrane resistance (mΩ)	*n*
Female	75.7 ± 3.4	− 74.2 ± 1.8	100.5 ± 5.9	10
Male	88.2 ± 4.9	− 77.1 ± 0.8	91.6 ± 4.6	10
*p* value	0.06	0.16	0.25	

It presents the passive membrane properties for whole-cell current clamp recordings of medium-spiny neurons in the nucleus accumbens shell

### Statistical analysis

Behavioral data collected from the ANY-maze software was analyzed using GraphPad Prism (GraphPad Software, LLC., version 9.3.1 for macOS). As appropriate, t-tests, one-way, or two-way ANOVAs were used. One-way ANOVAs were followed by Tukey’s multiple comparisons test. When variances were unequal between groups, a Welch’s *t*-test or Welch’s one-way ANOVA was used, assessed by an F-test to compare variances or a Brown–Forsythe test, respectively. Welch’s one-way ANOVAs were followed by Dunnett’s T3 multiple comparisons tests. For comparisons of data obtained from electrophysiology studies, two-way ANOVAS with Bonferroni post hoc tests were used. Data are expressed as the mean ± the standard error of the mean (SEM). Significance level was set at *p* ≤ 0.05 for all tests. For the behavioral portion, data from the two groups of females were combined, as there were no statistical differences between the groups at any point (data not shown).

## Results

### AAV expression and fiber optic placement

Fluorescently labeled cell bodies were found in the ILC (Fig. 2A), and fluorescently labeled axon terminals were found innervating the NAcSh (Fig. 2B). Furthermore, fiber optic placement was confirmed histologically (Fig. 2B), and only those mice that expressed correct placement of both the AAV and the fiber optic cannulae were included. Based on these criteria, three females and three males were excluded due to incorrect placement of either the AAV or fiber optic implants.

### Baseline

#### Baseline trial

During the initial baseline trial, mice were naive to the arena and therefore no zone should have held any significance. In the absence of stimulation, the number of entries into each zone was quantified, as well as the mean amount of time spent in each zone. At baseline, there were no significant differences in the mean number of entries into any zone between females (38.81 ± 3.12) and males (44.19 ± 1.20), as determined by a Welch’s t-test (*p* = 0.12). Similarly, the mean time in seconds (s) spent in any zone was not different between females (285.60 ± 11.98 s) and males (298.70 ± 16.44 s), determined by a Student’s t-test (*p* = 0.52).

### Acquisition

#### Comparison measurements within stimulus frequency, across stimulus frequency, and across sex

For the data presented below there were three major analyses. First, differences at a single stimulus frequency within sex, are denoted by an asterisk (*). The second compared within sex the effect of stimulus frequency, and is denoted by different letters (a, b, c). The third comparison determined sex differences within a single frequency (and sex differences are displayed by blue filled bars in the female dataset).

#### Sex differences in active zone entries during acquisition trials

**Fig. 3:**
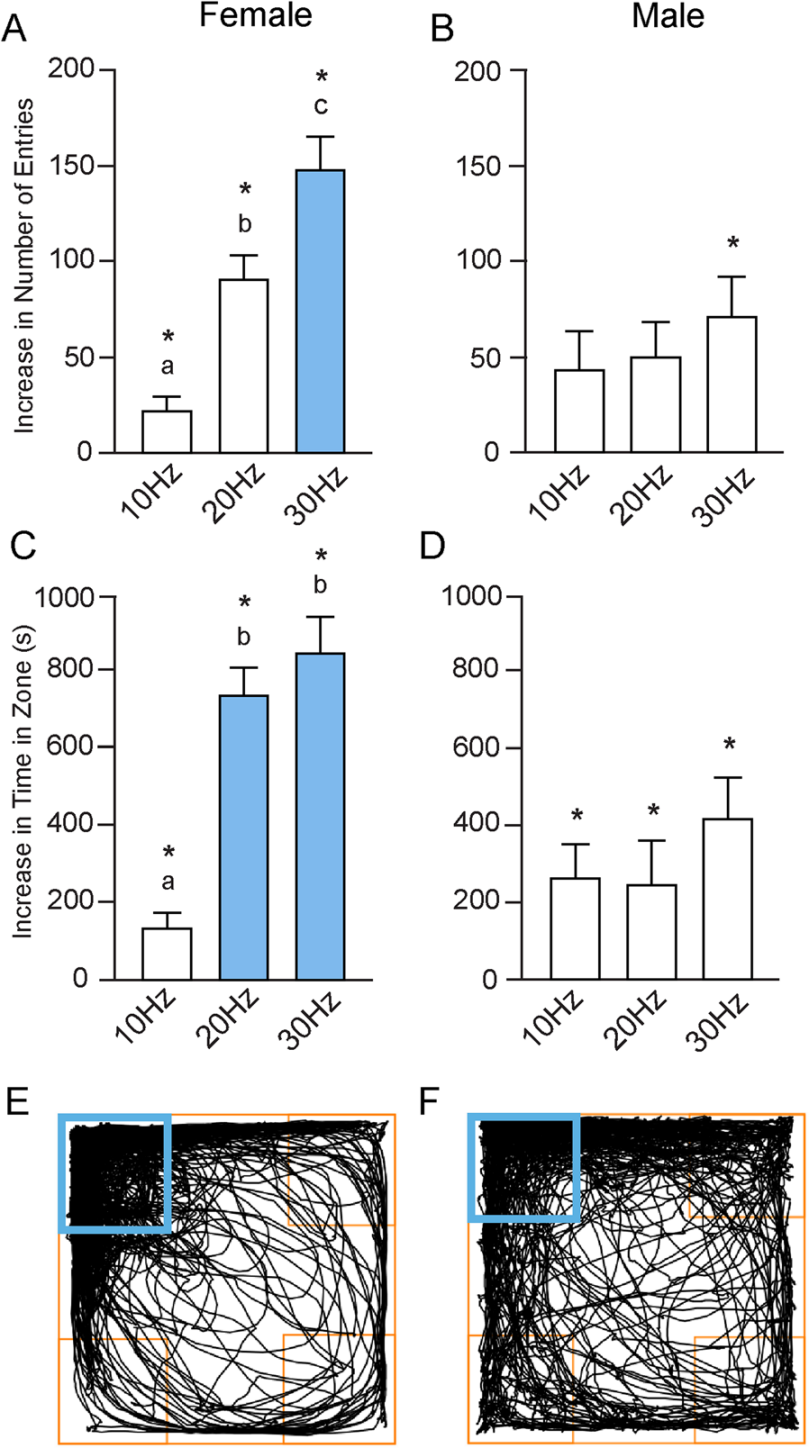
Sex differences in entries made and time spent in the acquisition zone during acquisition. **A** At all stimulation frequencies, the total number of entries into the acquisition zone were significantly greater than the average number of entries into the inactive zones (indicated by asterisks). There were also significant differences in the number of entries made into the active zone between 10 and 20 Hz, 10 Hz and 30 Hz, and 20 Hz and 30 Hz (denoted by different letters above each bar). At 30 Hz, there were significant differences between the sexes, shown as the colored bar. **B** Males entered the acquisition zone significantly more than the inactive zones only at 30 Hz. Males displayed no significant differences across frequencies in the number of entries into the acquisition zone. **C** In females, the total time spent in the acquisition zone was greater than the time spent in the inactive zones across all frequencies. There were also significant differences in the time spent in the acquisition zone between 10 and 20 Hz, and between 10 and 30 Hz. There were sex differences in the amount of time spent in the acquisition zone at 20 Hz and 30 Hz. **D** Males spent more total time in the acquisition zone versus the inactive zones at all frequencies. However, there were no significant differences in the time spent in the acquisition zone between any of the stimulation frequencies. Representative track plots illustrate travel throughout the duration of the 30-Hz acquisition trial for **E** females and **F** males. The acquisition zone is indicated by the blue square. Data presented as mean ± SEM

During the acquisition trial, we subtracted the average of the number of entries made into the three inactive zones from the number of entries into the acquisition zone to better assess the number of entries attributable to optogenetic stimulation (Fig. 3A, B). At each stimulation parameter we also compared the number of entries into the acquisition zone with the average number of entries into the inactive zones. Across all frequencies, female mice sought the acquisition zone significantly more than the inactive zones, determined by Student’s t-tests and indicated by asterisks on the graph. Specifically, during the 10-Hz trial, females entered the acquisition zone on average 62.67 ± 6.99 times, versus 39.92 ± 3.80 times (*p* = 0.009) on average for the inactive zones. At 20 Hz, the difference was 144.90 ± 13.88 times for the active zone versus an average of 53.50 ± 4.81 times (*p* < 0.0001) for the three inactive zones. At 30 Hz, the difference was 214.00 ± 14.36 times in comparison to 65.20 ± 10.04 (*p* < 0.0001).

For comparison of stimulus frequencies, a Welch’s one-way ANOVA was used, as a Brown–Forsythe test detected significant differences in the variance across stimulation frequencies (F_(2, 31)_ = 4.76; *p* = 0.02). The ANOVA indicated that there were significant differences in the number of entries made into the active zone between the stimulation parameters (W_(2, 17.28)_ = 30.70; *p* < 0.0001). A Dunnett’s T3 multiple comparisons test found that there were significant differences in the number of entries between 10 Hz (22.75 ± 6.70) and 20 Hz (91.42 ± 12.40; *p* = 0.0004), between 10 and 30 Hz (148.80 ± 16.44; *p* < 0.0001), and between 20 and 30 Hz (*p* = 0.03). Again, differences are denoted by different letters in Fig. 3A.

Performing the same analysis in males, we used Student’s t-tests to compare the number of entries into the acquisition zone with the average of the number of entries into the inactive zones. During the 10-Hz trial, there were no significant differences between entries into the acquisition zone (86.83 ± 20.81) and into the inactive zones (44.08 ± 2.72), but there was a trend toward significance (*p* = 0.06). The same held true during the 20-Hz trial, with no significant differences, but a trend toward significance, between entries into the acquisition zone (96.88 ± 21.42) versus the inactive zones (50.25 ± 6.74; *p* = 0.07). Only at 30 Hz did the males enter the acquisition zone (131.60 ± 22.29) significantly more than the inactive zones (60.22 ± 6.29; *p* = 0.01) (Fig. 3B) Regarding the effects of stimulus frequency, there were no significant differences in the number of entries made into the active zone across the 10-Hz (42.75 ± 20.22), 20 Hz (49.71 ± 17.98), and 30 Hz (71.33 ± 20.28) stimulation parameters (F_(2, 25)_ = 0.56; *p* = 0.58) (Fig. 3B).

We then sought to determine if there were sex differences in the increased number of entries into the acquisition zone at each stimulation frequency. Student’s t-tests indicated that there were no sex differences at 10 Hz (*p* = 0.36), and a trend toward significance at 20 Hz (*p* = 0.06). At 30 Hz stimulation, the difference in the number of entries made into the active zone was significantly greater in females than in males (*p* = 0.008), as indicated by the colored bar in Fig. 3A.

#### Sex differences in time in active zone during acquisition trials

As with the number of entries, we subtracted the average of the time spent in the three inactive zones from the time spent in the acquisition zone, and then compared the time in the active zone with the average time spent in the inactive zones across each stimulation parameter (Fig. 3C, D).

In females, at each stimulation frequency, mice spent significantly more time in the acquisition zone as compared with the inactive zones, determined by Student’s t-tests and indicated by asterisks on the graph. During the 10-Hz trial, females spent 403.30 ± 26.97 s in the acquisition zone, versus 271.50 ± 18.52 s (*p* = 0.0006) in the inactive zones. At 20 Hz the difference was 905.50 ± 56.69 s, in comparison to 168.90 ± 15.63 s (*p* < 0.0001), and at 30 Hz, 964.90 ± 75.87 s, versus 119.40 ± 20.97 s (*p* < 0.0001) (Fig. 3C).

For comparison across stimulus frequencies, a one-way ANOVA indicated that there were significant differences in the increase in time spent in the acquisition zone between the stimulation parameters (F _(2, 31)_ = 30.99; *p* < 0.0001) (Fig. 3C). Tukey’s multiple comparisons test found that the time spent in the active zone was different between 10 Hz (131.80 ± 40.30 s) and 20 Hz (736.60 ± 71.37 s; *p* < 0.0001) and between 10 and 30 Hz (845.50 ± 94.76 s; *p* < 0.0001). There were no statistical differences detected between 20 and 30 Hz. Again, the differences across frequencies are indicated by letters.

The same analyses were performed in males, using Student’s t-tests to compare the amount of time spent the acquisition zone, again with significant differences indicated by asterisks. During the 10-Hz trial, there were significant differences between the amount of time spent in the acquisition zone (499.70 ± 74.35 s) as compared with the inactive zones (234.00 ± 26.96 s; *p* = 0.005), as determined by a Welch’s t-test. The same was found during the 20-Hz trial, with male mice spending more time in the acquisition zone (505.70 ± 85.48 s) than in the inactive zones (256.30 ± 34.62 s; *p* = 0.02). Finally, a Student’s t-test indicated significant differences between the time spent in the acquisition zone (642.60 ± 85.85 s) as compared with the inactive zones (217.30 ± 33.58 s; *p* = 0.0009) during the 30-Hz trial (Fig. 3D).

Comparing across frequencies, a one-way ANOVA indicated that there were no significant differences in the increase in time spent in the acquisition zone between the stimulation frequency parameters (F _(2, 26)_ = 0.89; *p* = 0.42) (Fig. 3D). There were no significant differences between 10 Hz (265.70 ± 88.36 s) and 20 Hz (249.40 ± 114.50 s), between 10 and 30 Hz (425.30 ± 102.50 s), or between 20 and 30 Hz.

Finally, t-tests were utilized to determine if there were sex differences in the increase in time spent in the acquisition zone. During the 10-Hz trial, a Welch’s t-test indicated no differences between females and males (*p* = 0.18). However, Student’s t-tests indicated significant differences at both 20 (*p* = 0.001) and 30 Hz (*p* = 0.008), indicated by the colored bars in Fig. 3C. Representative 30-Hz track plots for female and male mice are shown in Fig. 3E,F.

#### Sex differences in bouts of optogenetic stimulation during acquisition trials

**Fig. 4:**
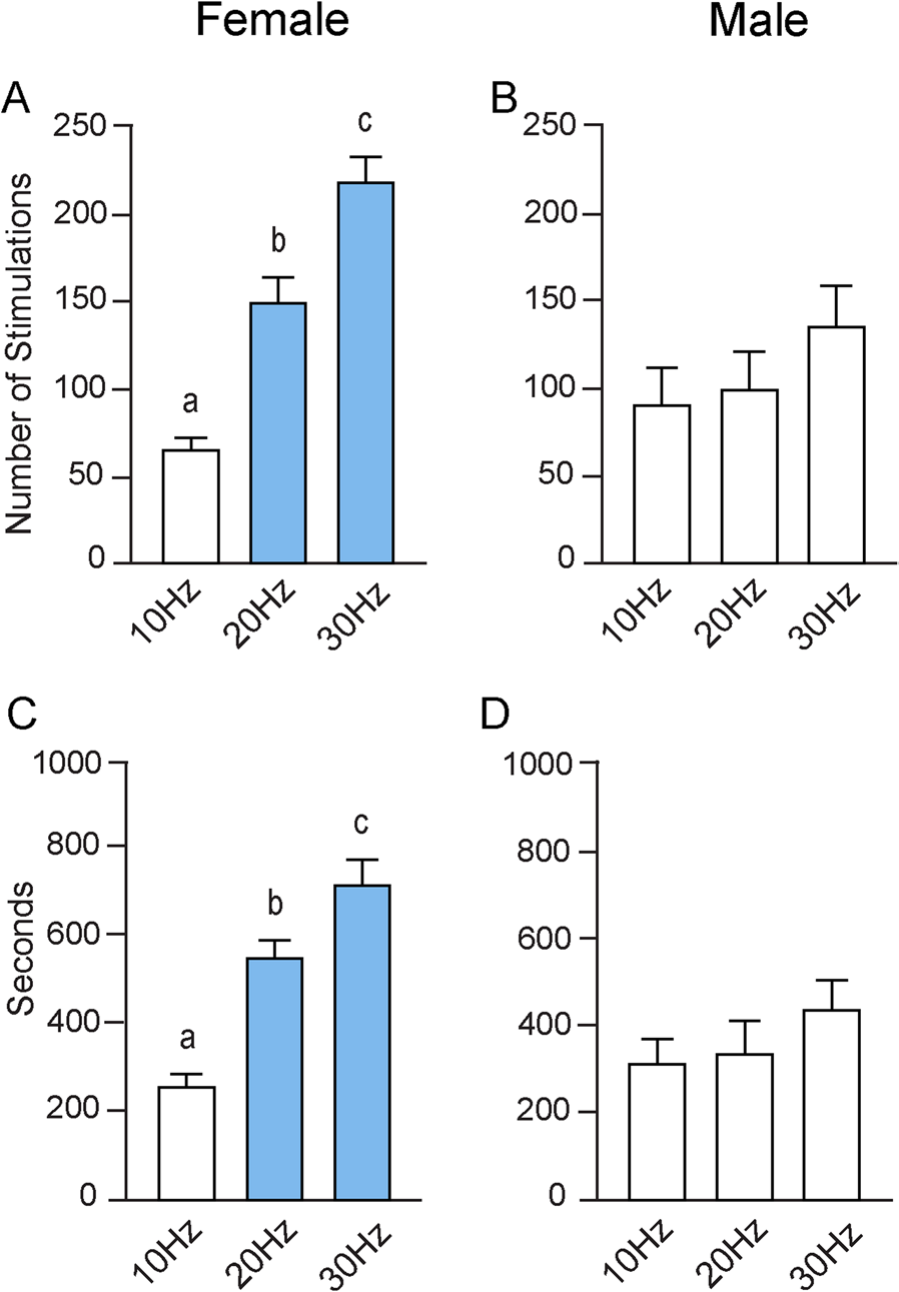
Sex differences in optogenetic stimulation during acquisition. **A** In females, there were significant differences in the number of optogenetic stimulation trains received during each of the three frequencies. Sex differences in the number of stimulation trains occurred at both 20 Hz and 30 Hz. **B** There were no differences in the number of optogenetic stimulation trains received by males across frequencies. **C** Across all stimulus conditions, there were significant differences in the amount of time female received optogenetic stimulation. At both 20 Hz and 30 Hz, there were also sex differences in optogenetic stimulation time. **D** In males, increasing stimulation frequency had no effect on the amount of time optogenetic stimulation was received

Similar to the results seen when analyzing the average number of entries, increasing the stimulation frequency significantly increased the number of stimulation trains females earned in the acquisition zone, as determined by a one-way ANOVA (F_(2, 31)_ = 42.68; *p* < 0.0001) (Fig. 4A). Tukey’s multiple comparisons test found a significant difference in the number of stimulations received between 10 Hz (65.50 ± 6.53) and 20 Hz (149.60 ± 13.57; *p* < 0.0001), between 10 and 30 Hz (218.80 ± 13.82; *p* < 0.001), and between 20 and 30 Hz (*p* = 0.0007). In males, however, increasing the stimulation did not affect the number of stimulations received in the active zone, as determined by a one-way ANOVA (F_(2, 26)_ = 1.19; *p* = 0.32) (Fig. 4B). There were no significant differences between 10 Hz (90.42 ± 21.01) and 20 Hz (99.25 ± 21.36), between 10 and 30 Hz (135.30 ± 22.49), or between 20 and 30 Hz. Sex differences in the number of stimulation trains received in the acquisition zone were found at both 20 Hz (*p* = 0.05) and 30 Hz (*p* = 0.005). In both cases, females received significantly more stimulation. A Welch’s t-test indicated that there were no differences between the number of stimulations received between the sexes at 10 Hz (*p* = 0.28).

#### Sex differences in total stimulation duration during acquisition trials

In females, increasing stimulation frequency significantly increased the total time stimulation was received, as determined by an ordinary one-way ANOVA (F_(2, 31)_ = 32.96; *p* < 0.0001) (Fig. 4C). Tukey’s multiple comparisons test found significant differences in the amount of time optogenetic stimulation was received between 10 Hz (260.00 ± 25.11 s) and 20 Hz (551.70 ± 39.32; *p* < 0.0001), between 10 and 30 Hz (716.60 ± 55.33 s; *p* < 0.0001), and between 20 and 30 Hz (*p* = 0.02). An ordinary one-way ANOVA indicated that increasing stimulation had no effect in males regarding the amount of time of optogenetic stimulation was received (F_(2, 26)_ = 1.10; *p* = 0.35) (Fig. 4D). There were no significant differences between 10 Hz (312.20 ± 55.98 s), 20 Hz (338.30 ± 71.50 s), or 30 Hz (437.70 ± 65.46). Comparing between sexes, a Student’s t-test indicated that at both 20 Hz (*p* = 0.01) and 30 Hz (*p* = 0.004), females received optogenetic stimulation for a longer amount of time than did males (Fig. 4C). There were no differences at 10 Hz (*p* = 0.41), as determined by a Welch’s t-test.

#### No sex differences in locomotor distance and speed during acquisition trials

**Fig. 5:**
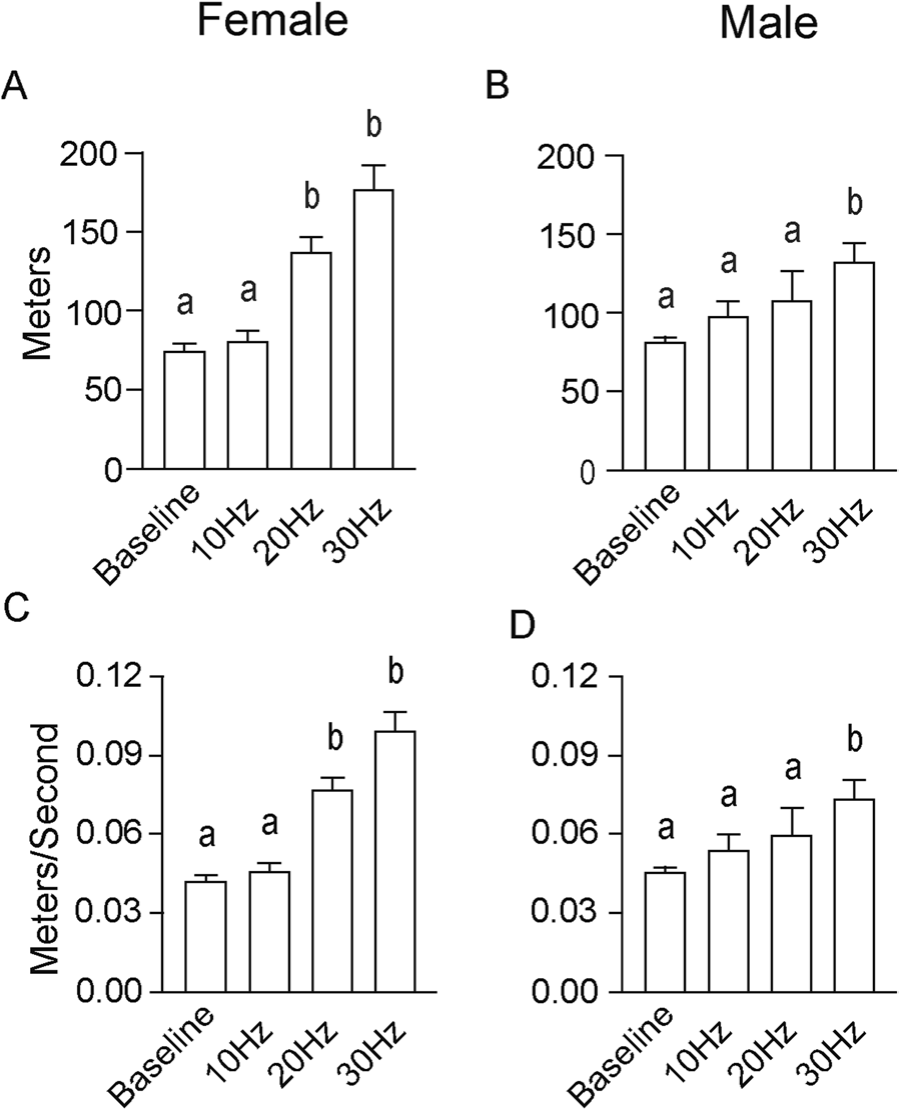
No sex differences in distance traveled or travel speed during acquisition. Heightening the stimulation frequency increased the mean distance traveled in **A** females and **B** males, though there were no sex differences at any frequency. Similarly, increasing stimulation frequency also increased the travel speed in both **C** females and **D** males, again without differences between the sexes

During all acquisition trials, the mean distance traveled, in meters (m) and mean travel speed, in meters per second (m/s), were analyzed. There were no significant differences in either the distance traveled or rate of travel between females and males during any trial, as determined by Student’s t-tests (Fig. 5). At baseline, there were no differences in distance traveled between females (74.90 ± 4.99 m; Fig. 5A) and males (81.00 ± 2.92 m; *p* = 0.30; Fig. 5B), nor were there differences in the travel speed between females (0.041 ± 0.003 m/s; Fig. 5C) and males (0.045 ± 0.002 m/s; *p* = 0.28; Fig. 5D). During the 10-Hz acquisition trial, there were no significant differences in the distance traveled between females (80.92 ± 6.99 m) and males (97.83 ± 9.93 m; *p* = 0.18). Similarly, the travel speed was not different between females (0.045 ± 0.004 m/s) and males (0.054 ± 0.006 m/s; *p* = 0.18). During the 20-Hz acquisition trial, again there were no significant differences in the distance traveled between females (137.10 ± 9.67 m) and males (108.50 ± 18.02 m; *p* = 0.15). Again, the travel speed was not different between females (0.076 ± 0.005 m/s) and males (0.060 ± 0.010 m/s; *p* = 0.15). Finally, during the 30-Hz acquisition trial, there were no differences in the distance traveled between females (177.10 ± 14.61 m) and males (147.30 ± 12.06 m; *p* = 0.16). The travel speed was not different between females (0.098 ± 0.008 m/s) and males (0.082 ± 0.007 m/s; *p* = 0.16).

In contrast, increasing the stimulation frequency significantly increased both the mean distance traveled and the mean travel speed *within* both sexes, as compared with the baseline trial. In females, a Welch’s one-way ANOVA was used, as a Brown–Forsythe test detected significant differences in the variance of one or more groups (F_(3, 46)_ = 3.99; *p* = 0.01). The Welch’s ANOVA indicated that there were significant differences in the distance traveled between the stimulation parameters (W_(3, 21.90)_ = 25.50; *p* < 0.0001). A Dunnett’s T3 multiple comparisons test found significant differences between baseline and 20 Hz (*p* = 0.0001), between baseline and 30 Hz (*p* = 0.0002), between 10 and 20 Hz (*p* = 0.0008), and between 10 and 30 Hz (*p* = 0.0003) (Fig. 5A). Similarly, a Welch’s one-way ANOVA was used to analyze differences in the travel speed in females, as a Brown–Forsythe test detected significant differences in the variance of one or more groups (F_(3, 46)_ = 3.95; *p* = 0.01). The ANOVA indicated that there were statistically significant differences in the travel speed between the stimulation parameters (W_(3, 21.89)_ = 21.75; *p* < 0.0001). A Dunnett’s T3 multiple comparisons test found that the travel speed was significantly different between baseline and 20 Hz (*p* = 0.0002), between baseline and 30 Hz (*p* = 0.0002), between 10 and 20 Hz (*p* = 0.0009), and between 10 and 30 Hz (*p* = 0.0003) (Fig. 5C).

Males responded similarly to increases in stimulation frequency. A Welch’s one-way ANOVA was used to analyze the distance traveled, as a Brown–Forsythe test detected significant differences in the variance of one or more groups (F_(3, 41)_ = 4.08; *p* = 0.01). The Welch’s ANOVA indicated that there were significant differences in the distance traveled between the stimulation parameters (W_(3, 15.09)_ = 5.08; *p* = 0.01). A Dunnett’s T3 multiple comparisons test found significant differences between baseline and 30 Hz (*p* = 0.03) (Fig. 5B). Again, a Welch’s one-way ANOVA was used to analyze differences in the travel speed in males, as a Brown–Forsythe test detected significant differences in the variance of one or more groups (F_(3, 41)_ = 4.11; *p* = 0.01). The ANOVA indicated that there were significant differences in the travel speed between the stimulation parameters (W_(3, 15.07)_ = 5.01; *p* = 0.01). A Dunnett’s T3 multiple comparisons test found significant differences between baseline and 30 Hz (*p* = 0.03) (Fig. 5D).

### Reversal

#### No sex differences in active zone entries during reversal trials

**Fig. 6:**
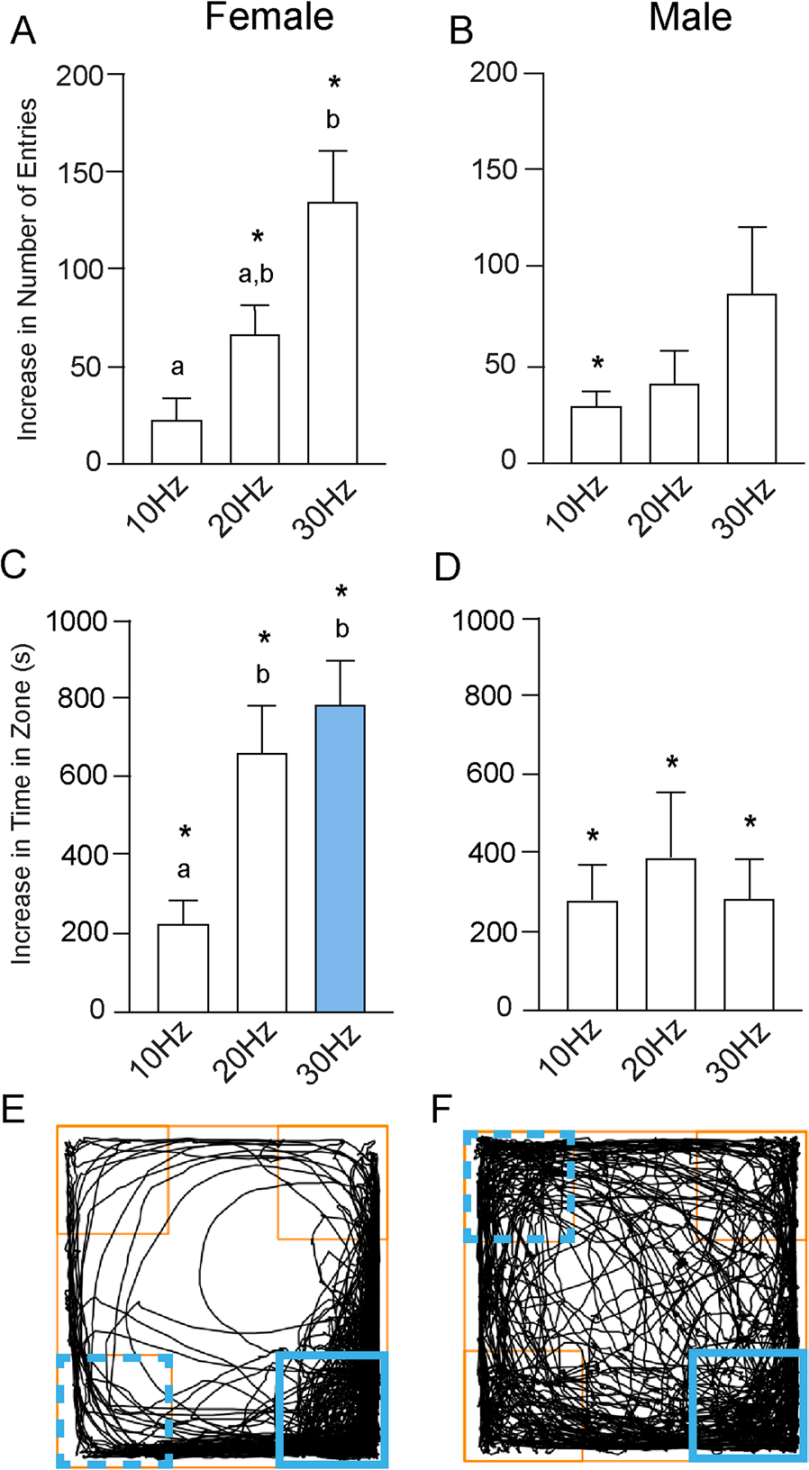
Entries made and sex differences in time spent in the reversal zone. **A** Females entered the reversal zone versus the previously active zone a greater number of times at 20 Hz and 30 Hz. The number of entries into the reversal zone was also greater at 30 Hz than 10 Hz. **B** Males entered the reversal zone significantly more only at 10 Hz and showed no differences in entries across stimulation frequencies. **C** Females spent more time in the reversal zone versus the acquisition zone at all three stimulus frequencies. They also spent more time in the reversal zone at 20 Hz and 30 Hz in comparison to 10 Hz. At 30 Hz, there was a sex difference in the time spent in the reversal zone. **D** In males, there were significant differences in the time spent in the reversal zone as compared with time spent in the acquisition at all stimulus frequencies. However, there were no differences across frequency. Representative track plots illustrate travel throughout the duration of the 30-Hz reversal trial for both **E** females and **F** males. The reversal zone is indicated by the solid blue square, and the previous acquisition zone is indicated by the dashed blue square

During the reversal day trials, to better assess the difference in the number of entries made into the previous day’s active zone (“Zone 1”) versus the reversal day’s active zone (“Zone 2”), we subtracted the average of the entries made into Zone 1 from the average of the entries made into Zone 2 (Fig. 6A, B). This aided in analyzing if the mice were able to learn to seek the new active zone, and to begin to parse out behavioral strategies involved.

In females, a Welch’s *t*-test indicated no differences in the number of entries between Zone 2 (73.50 ± 13.06) and Zone 1 (50.33 ± 6.09; *p* = 0.13) in the 10-Hz trial. However, Welch’s *t*-tests indicated that at 20 Hz females entered Zone 2 136.80 ± 17.23 times, versus 70.42 ± 7.17 (*p* = 0.003) into Zone 1, and at 30 Hz, females entered Zone 2 194.30 ± 24.61 times, as compared with 59.30 ± 7.34 (*p* = 0.0003) entries into Zone 1 (Fig. 6A).

Comparing across frequencies, a Welch’s one-way ANOVA was used, as a Brown–Forsythe test detected significant differences in the variance across stimulation frequencies (F_(2, 31)_ = 6.17; *p* = 0.006). The ANOVA indicated that there were statistically significant differences in the number of entries into Zone 2 between the stimulation parameters (W_(2, 17.78)_ = 8.82; *p* = 0.002) (Fig. 6A). A Dunnett’s T3 multiple comparisons test found significant differences in the number of entries into Zone 2 between 10 Hz (23.17 ± 10.38) and 30 Hz (135.00 ± 26.10; *p* = 0.005), but not between 10 and 20 Hz (66.42 ± 15.06) or between 20 and 30 Hz (Fig. 6A).

Repeating these analyses in males, Student’s t-tests indicated significant differences between the number of entries made into Zone 2 versus Zone 1 during both the 10-Hz and 30-Hz trials. At 10 Hz, males entered Zone 2 an average of 77.55 ± 7.42 times, versus 47.91 ± 4.96 (*p* = 0.003) entries into Zone 1. During the 30-Hz trial there was a trend toward significance; males entered Zone 2 an average of 139.40 ± 31.55 times, as compared with 71.78 ± 8.56 entries into Zone 1 (*p* = 0.06). There were no differences in the number of entries between Zone 2 (99.33 ± 23.05) and Zone 1 (57.38 ± 8.31) during the 20-Hz trial (*p* = 0.14).

To compare across frequencies, a Welch’s one-way ANOVA was used, as a Brown–Forsythe test detected significant differences in the variance across stimulation frequencies (F_(2, 23)_ = 5.86; *p* = 0.009). The ANOVA indicated that there were not statistically significant differences in the number of entries into the new active zone between the stimulation frequency parameters (W_(2, 10.56)_ = 1.77; *p* = 0.22) (Fig. 6B). There were no significant differences between 10 Hz (29.64 ± 8.01) and 20 Hz (41.50 ± 16.66), between 10 and 30 Hz (89.11 ± 30.44), or between 20 and 30 Hz. Student’s t-tests indicated that, unlike during acquisition trials, there were no sex differences in the number of entries at 10 Hz (*p* = 0.63), 20 Hz (*p* = 0.32), or 30 Hz (*p* = 0.27).

#### Sex differences in time in active zone during reversal trials

We subtracted the average amount of time spent in Zone 1 from the average amount of time spent in Zone 2 during the reversal day to assess the difference in the amount of time spent in the new active zone as compared with the previous active zone (Fig. 6C, D). Comparing within frequencies in females, Welch’s t-tests found significant differences between the amount of time spent in Zone 2 as compared with Zone 1 at all stimulation frequencies. During the 10-Hz trial, females spent 450.00 ± 46.24 s in Zone 2, versus 221.10 ± 19.20 s in Zone 1 (*p* = 0.0004). During the 20-Hz trial, mice spent 865.50 ± 91.46 s in Zone 2, versus 203.90 ± 34.76 s in Zone 1 (*p* < 0.0001). Finally, during the 30-Hz trial, females spent 923.40 ± 83.78 s in Zone 2, as compared with 136.20 ± 33.73 s in Zone 1 (*p* < 0.0001) (Fig. 6C).

Comparing across frequencies, a one-way ANOVA indicated significant differences in the amount of time spent in Zone 2 across stimulation frequencies (F_(2, 31)_ = 8.298; *p* = 0.0008) (Fig. 6C). Tukey’s multiple comparisons test found significant differences between 10 Hz (228.90 ± 55.68 s) and 20 Hz (661.60 ± 117.80 s; *p* = 0.008) and between 10 and 30 Hz (787.20 ± 111.80; *p* = 0.001). There were no differences between 20 and 30 Hz (Fig. 6C).

In males, Welch’s t-tests also indicated significant differences in the amount of time spent in Zone 2 as compared with Zone 1 at all frequencies. During the 10-Hz trial, males spent 560.80 ± 62.35 s in Zone 2 compared with 273.40 ± 37.02 in Zone 1 (*p* = 0.0008). During the 20-Hz trial, males spent 622.80 ± 135.60 s in Zone 2, versus 226.00 ± 40.46 s in Zone 1 (*p* = 0.03), and during the 30-Hz trial, mice spent 501.80 ± 88.62 s in Zone 2 as compared with 220.50 ± 26.97 s in Zone 1 (*p* = 0.01) (Fig. 6D).

Comparing across frequencies, a one-way ANOVA indicated no significant differences in the increase in the amount of time spent in Zone 2 over that spent in Zone 1 across stimulation frequencies (F_(2, 23)_ = 0.30; *p* = 0.74). There were no significant differences between 10 Hz (287.50 ± 80.52 s) and 20 Hz (396.80 ± 158.20 s), between 10 and 30 Hz (281.30 ± 101.80 s), or between 20 and 30 Hz (Fig. 6D).

Student’s t-tests indicated no differences in the increase in the amount of time spent in Zone 2 between females and males during the 10-Hz (*p* = 0.55) or the 20-Hz (*p* = 0.21) trials. Females spent significantly more time in the new active zone than males did during the 30-Hz trial (*p* = 0.004) (Fig. 6C).

#### No sex differences in bouts of optogenetic stimulation during reversal trials

**Fig. 7:**
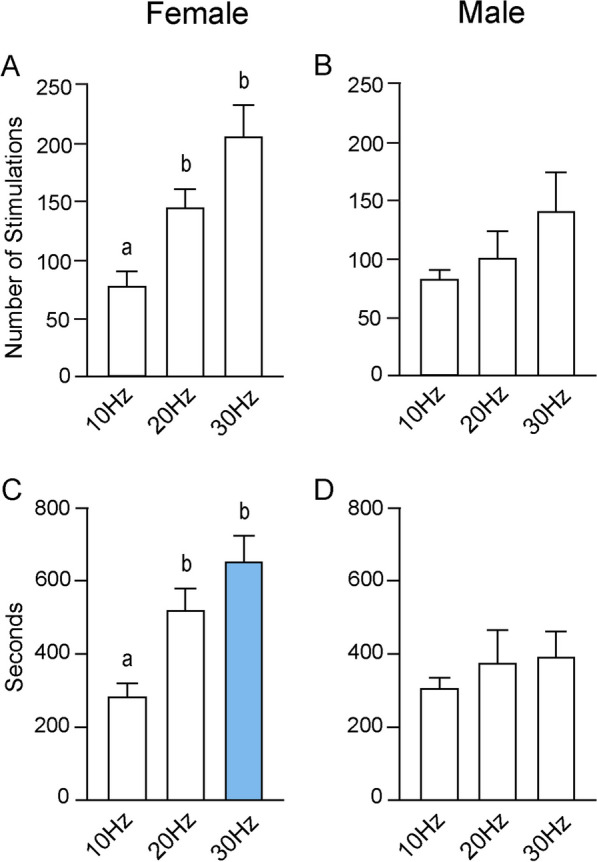
Optogenetic stimulation during reversal trials. **A** In females, there were significant differences in the number of optogenetic stimulation trains received during reversal trials between 10 Hz versus 20 Hz and 30 Hz. **B** In males, increasing the optogenetic stimulation frequency did not increase the number of stimulations. **C** Increasing stimulation frequency in females resulted in differences in the mean amount of time optogenetic stimulation was received, again between 10 Hz versus 20 Hz and 30 Hz. At 30 Hz stimulation, females received significantly more optogenetic stimulation than did males. **D** In males, there were no significant differences in mean time optogenetic stimulation

A one-way ANOVA indicated that increasing stimulation frequency during the reversal trials significantly increased the number of stimulations females received in the reversal zone (F_(2, 30)_ = 12.51; *p* = 0.0001) (Fig. 7A). Tukey’s multiple comparisons test found significant differences in the number of stimulations received between 10 Hz (77.75 ± 12.24) and 20 Hz (144.30 ± 16.27; *p* = 0.02) and between 10 and 30 Hz (205.80 ± 25.71; *p* < 0.0001). There was a trend toward significance between 20 and 30 Hz (*p* = 0.06). Performing the same analysis in males indicated that increasing stimulation frequency during the second day of testing significantly had no effect on the number of stimulations received in the reversal zone (F_(2, 23)_ = 1.95; *p* = 0.16) (Fig. 7B). Tukey’s multiple comparisons test found no significant differences in the number of stimulations received between 10 Hz (84.64 ± 6.84) and 30 Hz (141.90 ± 32.64; *p* = 0.14), between 10 and 20 Hz (101.70 ± 37.42; *p* = 0.86) or between 20 and 30 Hz (*p* = 0.48). There were no differences in the number of stimulations received in the reversal zone between females and males at 10 Hz (*p* = 0.64), 20 Hz (*p* = 0.15), or 30 Hz (*p* = 0.14) stimulation parameters.

#### Sex differences in total stimulation duration during reversal trials

In females, a one-way ANOVA indicated that increasing stimulation frequency resulted in significant differences in average amount of time optogenetic stimulation was received in the reversal zone (F_(2, 30)_ = 12.02; *p* = 0.0001) (Fig. 7C). Tukey’s multiple comparisons test found that there were significant differences in the amount of time that optogenetic stimulation was received in the active zone between 10 Hz (284.90 ± 36.68 s) and 20 Hz (522.50 ± 56.30 s; *p* = 0.007) and between 10 and 30 Hz (656.00 ± 69.42 s; *p* = 0.0001), but not between 20 and 30 Hz.

In males, a one-way ANOVA found no significant differences in the time optogenetic stimulation was received in the reversal zone across stimulation parameters (F_(2, 23)_ = 0.67; *p* = 0.52) (Fig. 7D). There were no significant differences between 10 Hz (308.90 ± 24.52 s) and 20 Hz (376.40 ± 86.74 s), between 10 and 30 Hz (389.30 ± 68.82 s), or between 20 and 30 Hz. Finally, there were sex differences in the amount of time optogenetic stimulation was received in the reversal zone only at 30 Hz (*p* = 0.01), but not at 10 Hz (*p* = 0.60) or 20 Hz (*p* = 0.16).

#### No sex differences in locomotor distance and speed during reversal trials

**Fig. 8:**
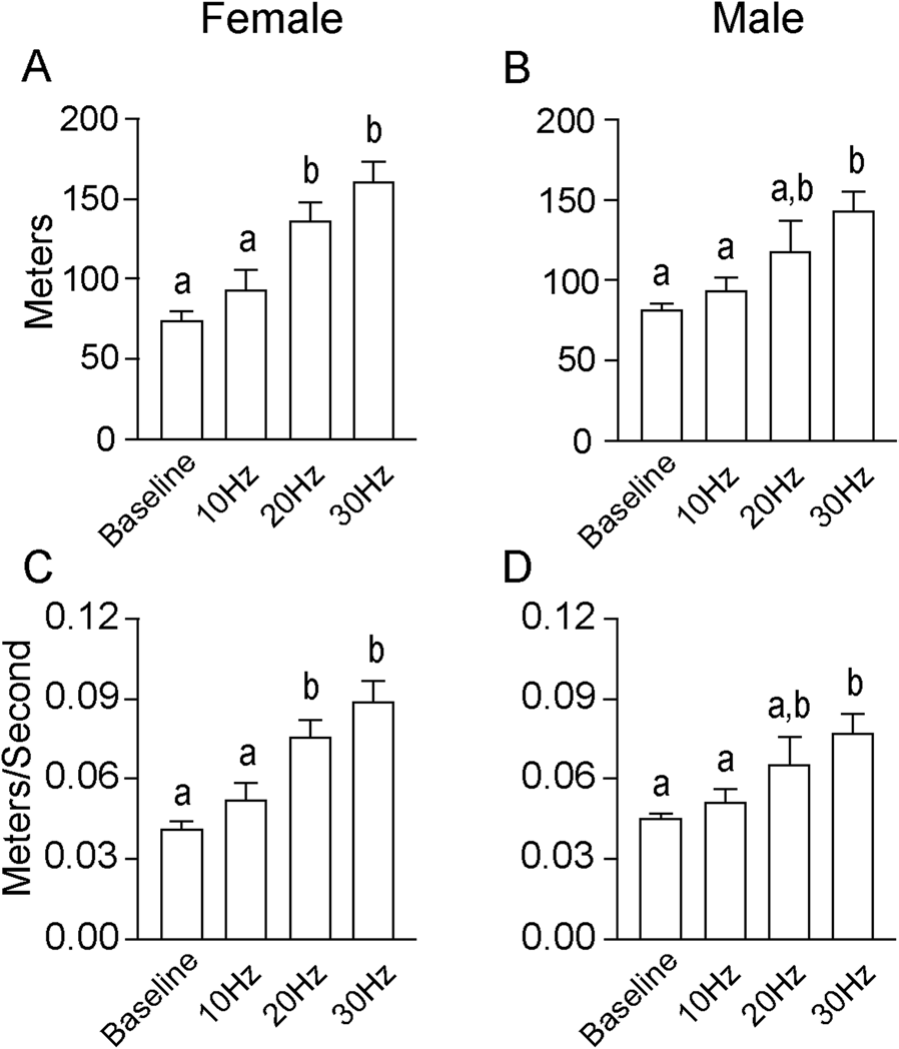
Distance traveled and travel speed during reversal trials. As with the acquisition trials, heightening the stimulation frequency increased the mean distance traveled in both **A** females and **B** males, without any differences between the sexes. Similarly, increasing frequency stimulation also increased the travel speed in **C** females and **D** males, without sex differences

As during the acquisition trials, the mean distance traveled and the mean travel speed was analyzed during the reversal trials, and again, there were no significant differences between the sexes in either parameter at any stimulation frequency (Fig. 8). During the 10-Hz reversal trial, there were no significant differences in the distance traveled between females (94.26 ± 12.07 m; Fig. 8A) and males (94.30 ± 6.91 m; *p* = 0.99; Fig. 8B), nor were there differences in travel speed between females (0.052 ± 0.007 m/s; Fig. 8C) and males (0.052 ± 0.004 m/s; *p* = 0.99; Fig. 8D). There were no differences in the distance traveled between females (137.00 ± 11.65 m) and males (118.50 ± 18.41 m) during the 20-Hz reversal trial (*p* = 0.39). Similarly, the mean speed was not different between females (0.076 ± 0.006 m/s) and males (0.066 ± 0.010 m/s; *p* = 0.39). Finally, during the 30-Hz reversal trial, there were no differences in the mean distance traveled between females (161.30 ± 12.72 m) and males (143.10 ± 11.62 m; *p* = 0.31). Similarly, there were no differences in the travel speed between females (0.090 ± 0.007 m/s) and males (0.079 ± 0.006 m/s; *p* = 0.31).

Similar to the results during the acquisition trials, increasing the stimulation frequency during the reversal trials increased both the mean distance traveled and the mean travel speed *within* both sexes as compared with the baseline measurements. In females, an ordinary ANOVA indicated that there were significant differences in the distance traveled between the stimulation parameters (F_(3, 46)_ = 15.32; *p* < 0.0001). A Tukey’s multiple comparisons test found significant differences in the distance traveled between baseline and 20 Hz trials (*p* = 0.0002), between baseline and 30 Hz (*p* < 0.0001), between 10 and 20 Hz (*p* = 0.02), and between 10 and 30 Hz (*p* = 0.0003) (Fig. 8A). Similarly, an ordinary one-way ANOVA indicated that there were significant differences in the travel speed between the stimulation parameters (F_(3, 46)_ = 15.35; *p* < 0.0001). A Tukey’s multiple comparisons test found that the travel speed was significantly different between baseline and 20 Hz (*p* = 0.0002), between baseline and 30 Hz (*p* < 0.0001), between 10 and 20 Hz (*p* = 0.02), and between 10 and 30 Hz (*p* = 0.0003) (Fig. 8C).

In males, a Welch’s one-way ANOVA was used, as a Brown–Forsythe test detected significant differences in the variance of one or more groups (F_(3, 38)_ = 5.86; *p* = 0.002). The Welch’s ANOVA indicated that there were statistically significant differences in the mean distance traveled between the stimulation parameters (W_(3, 13.09)_ = 9.56; *p* = 0.001). A Dunnett’s T3 multiple comparisons test found significant differences between baseline and 30 Hz (*p* = 0.003) and between 10 and 30 Hz (*p* = 0.01) (Fig. 8B). Finally, a Welch’s one-way ANOVA was used to analyze differences in the travel speed in males, as a Brown–Forsythe test detected significant differences in the variance of one or more groups (F_(3, 38)_ = 5.72; *p* = 0.003). The ANOVA indicated that there were significant differences in the travel speed between the stimulation parameters (W_(3, 13.07)_ = 9.37; *p* = 0.001). A Dunnett’s T3 multiple comparisons test found differences between baseline and 30 Hz (*p* = 0.003) and between 10 and 30 Hz (*p* = 0.02) (Fig. 8D) (Additional files 1, 2, 3).

### Electrophysiology

#### Sex differences in synaptic strength and intrinsic excitability

**Fig. 9:**
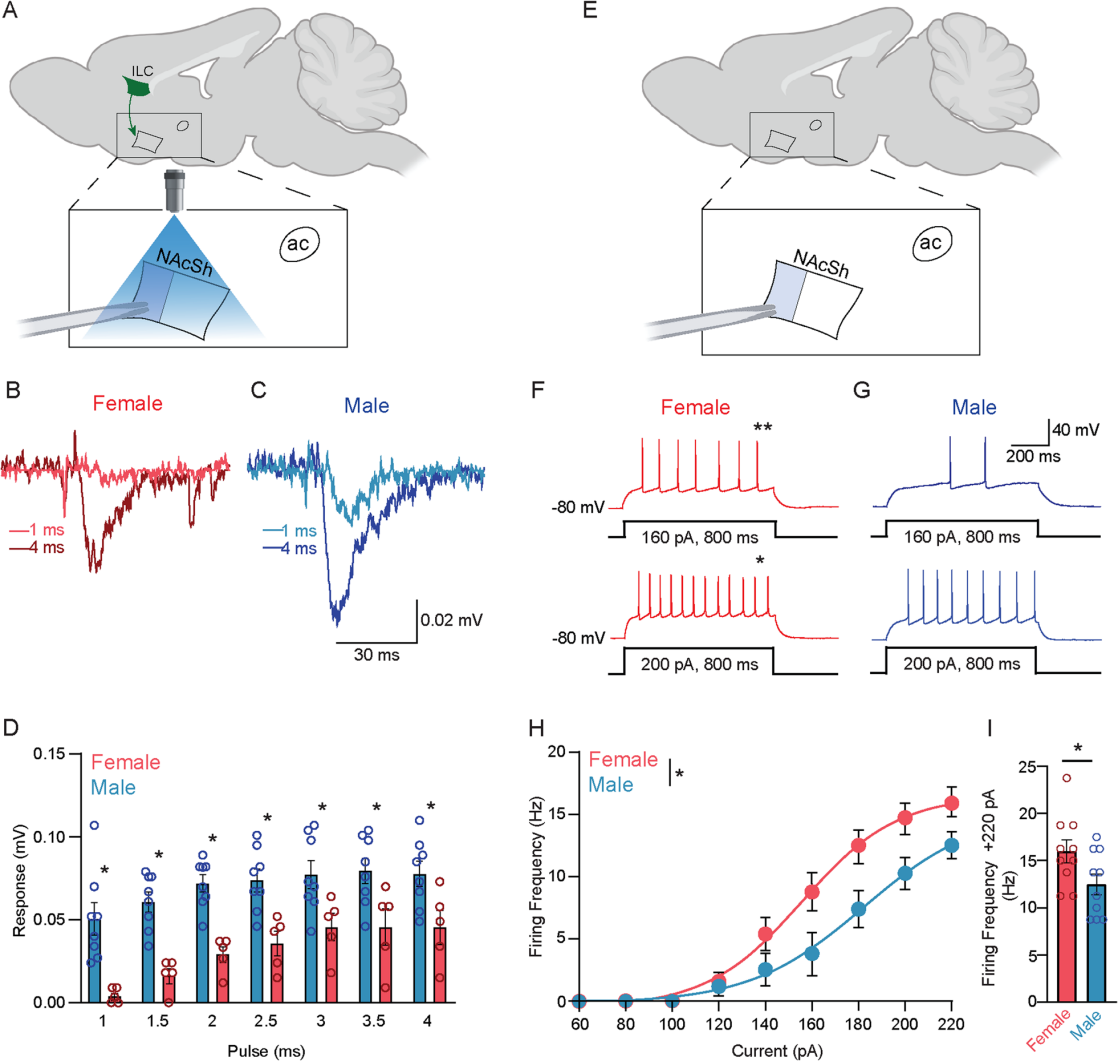
Males exhibit stronger ILC-NAcSh glutamatergic neurotransmission, while females display increased MSN intrinsic excitability. **A** Neurons in the infralimbic cortex (ILC) in both female and male mice were transfected with an eYFP-labeled AAV expressing channelrhodopsin, and the terminals in the shell of the nucleus accumbens (NAcSh) were optogenetically stimulated ex vivo. The shaded area in the enlarged box indicates recording area. Representative traces of the measured optogenetic local field potentials (oLFPs) in both female (**B**) and male (**C**) mice are presented at light stimulation durations of 1 ms (ms) (**B**: female, light red; C: male, light blue) and 4 ms (**B** female, dark red; **C** male, dark blue). **D** At all stimulus frequencies, males (blue) exhibited a significantly greater glutamatergic response (*p* = 0.002) than females (red). **E** Whole-cell current clamp recordings were measured in medium-spiny neurons (MSNs) in NAcSh. The shaded area in the enlarged box indicates recording area. Representative current clamp recordings of female (**F**) and male **G** NAcSh MSNs at 160 pA (top) and 200 pA (bottom) depolarizations are presented. Input current elicited significantly more action potentials in females than in males at 160 pA (**F** top) and 200 pA (**F** bottom), indicated by the asterisk. **H** Females (red) exhibited significantly greater intrinsic firing frequencies than males (blue) in response to current injection (*p* = 0.0489), and post hoc analysis indicated significant differences at 160 pA through 200 pA (*p* < 0.05). **I** Females (red) had significantly increased NAcSh MSN action potential firing frequency at maximum current injection (+ 220 pA) compared to males (blue) (*p* = 0.0435). NAcSh, nucleus accumbens shell; ac, anterior commissure; AAV, AAV adeno-associated virus; ILC infralimbic cortex

Heightened optogenetic self-stimulation behavior in females could be due to sex differences in synaptic strength and/or in intrinsic excitability. First, we examined the strength of the ILC-NAcSh connection via optogenetic local field potentials (oLFP) using ex vivo slice recordings (Fig. 9A). Paradoxical to our behavioral measures, at all tested stimulus durations, male animals exhibited significantly increased oLFP strength in comparison to females (two-way ANOVA, F_(1,11)_ = 15.53, *p* = 0.002) (Fig. 9B–D). To assess whether the sex differences in glutamatergic synaptic strength were related to differences in MSN excitability, current clamp recordings of NAcSh MSNs were performed in a separate group of animals (Fig. 9E). Contrary to the sex differences in oLFP glutamatergic synaptic strength (males > females), female NAcSh MSN neuronal excitability was greater compared to males across a current injection stimulus response curve (two-way ANOVA, F_(1,18)_ = 4.46, *p* = 0.0489) (Fig. 9F–H). The maximum firing frequency elicited at + 220 pA current injection was also significantly higher in females compared to males (16.0 ± 1.2 Hz vs 12.5 ± 1.1 Hz, unpaired t-test, *p* = 0.0435) (Fig. 9I).

**Fig. 10:**
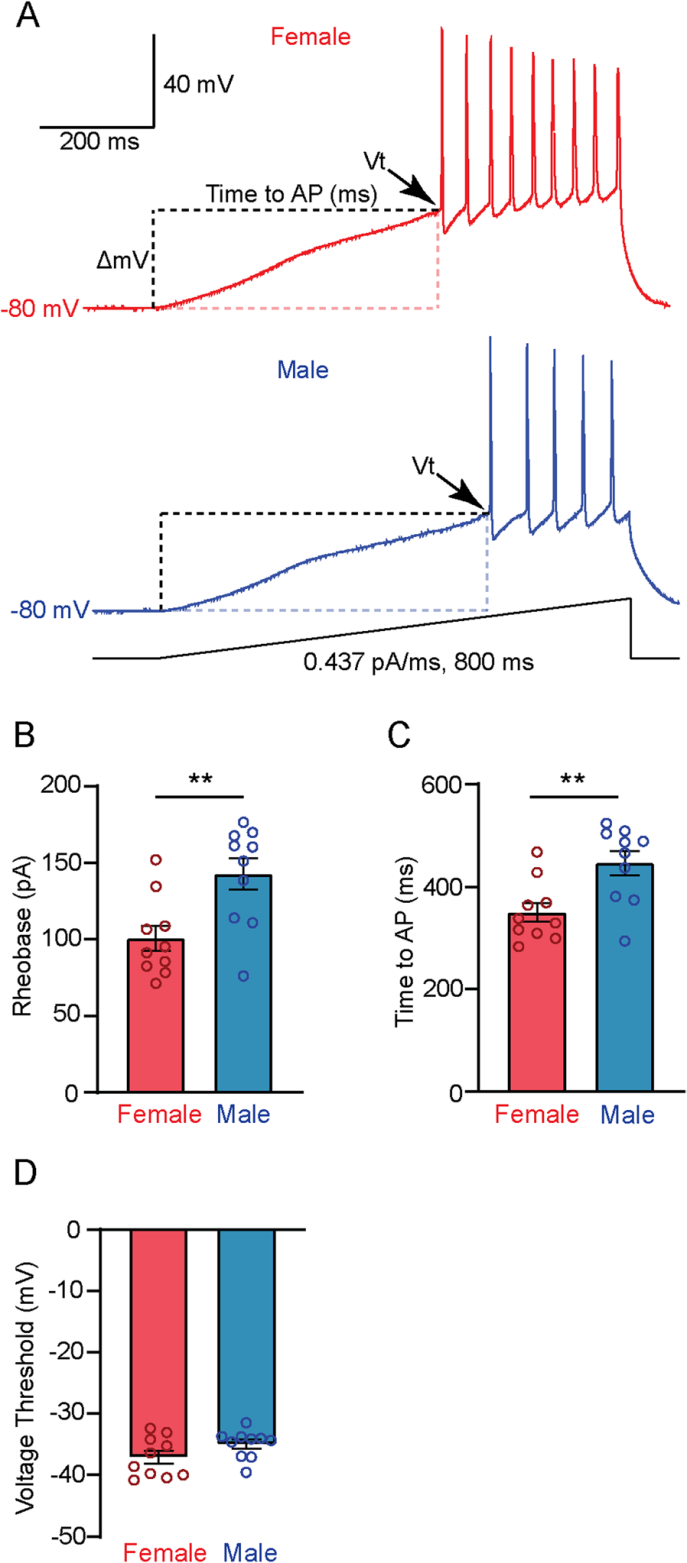
Females have a lower rheobase and shorter time to initiate an action potential compared to males. **A** Representative ramp current injection traces for females (red, top) and males (bottom, blue). **B** Females (red) have a significantly lower rheobase compared to males (blue) (*p* = 0.005). **C** Females (red) have a significantly shorter time to initiate an action potential compared to males (blue) (*p* = 0.005). **D** There is no apparent sex difference in voltage threshold to firing an AP between females (red) and males (blue) (*p* = 0.1064)

Further analysis from the ramp protocol (Fig. 10A–D) revealed sex differences in rheobase, where females required less current injection to fire an action potential (100.6 ± 8.1 pA vs 142.3 ± 10.3 pA, unpaired t-test, *p* = 0.005) (Fig. 10B). Female NAcSh MSNs also had a shorter duration in time from onset of current injection to firing an action potential (350.3 ± 18.6 ms vs 446.5 ± 23.6 ms, unpaired *t*-test, *p* = 0.005) (Fig. 10C). Voltage threshold to firing an action potential (Vt) was not significantly different between females and males (− 37.1 ± 1.0 mV vs − 35.0 ± 0.7 mV, unpaired t-test, *p* = 0.1064) (Fig. 10D). We also found no sex differences in passive NAcSh MSN membrane properties (capacitance, resting membrane potential, membrane resistance and hyperpolarizing input resistance) (Table 2). Taken together the data indicate that while glutamatergic synaptic inputs from the ILC to the NAcSh may be stronger in males than females, the heightened intrinsic excitability of female NAcSh neurons promotes the observed sex differences in motivated behavior.

## Discussion

Here we describe three principal findings. First, females are more behaviorally sensitive to stimulation of the ILC to NAcSh circuit than males. As stimulation frequency increased, so did female reward-seeking behavior, on both acquisition and reversal days. During reversal day trials, female mice were able to rapidly learn the new location, while retaining the behavioral strategy necessary to receive stimulation. In contrast, while males displayed increased time in the active zone for both acquisition and reversal across multiple frequencies, optogenetic stimulation was much less effective in driving motivated behavior. Increasing stimulation frequency did not significantly increase reward-seeking behavior during acquisition day trials. In addition, the observed sex differences decreased on reversal days, suggesting that males need more exposure to the stimulation to refine their strategy. These data support previous work indicating that optogenetic stimulation of the glutamatergic ILC-NAcSh circuit drives reward seeking, and that this stimulation provides positive reward valence [10], and are consistent with previous data indicating that ILC-NAcSh stimulation can reinforce a pattern of motor behavior that precedes it, thus leading to its repetition [18]. Second, there are sex differences in glutamatergic synaptic strength in the ILC-NAcSh circuit. Optogenetic local field potentials from neurons in the NAcSh were significantly greater in males as compared to females at all stimulation durations. These data are paradoxical to the observed behavior. Third, we found a sex difference in the intrinsic excitability of NAcSh neurons. In this instance, females displayed significantly greater excitability than males, consistent with the behavioral observations.

In the behavioral task, the acquisition trial allowed us to understand: (1) if stimulation of the ILC-NAcSh pathway was reinforcing, (2) if the mice learned to seek the stimulation, and (3) the behavioral strategy or strategies utilized. During acquisition trials, female mice were particularly attuned to the rewarding properties of ILC-NAcSh stimulation. As optogenetic stimulation frequency increased, so did the behavioral output. The number of entries into the active zone increased significantly between all stimulation parameters (Fig. 3A), resulting in a corresponding increase in the number of stimulation trains received (Fig. 4A). Additionally, the difference between the number of entries into the active zone versus into the inactive zones increased significantly with increasing stimulation frequency, suggesting that higher stimulation drove increased reinforcement of reward-seeking behavior. While there was an increase in the amount of time spent in the active zone between 10 and 20 Hz, no such increase was seen between 20 and 30 Hz (Fig. 3C). Interestingly, there were differences in the amount of time optogenetic stimulation was received between the two higher stimulation parameters (Fig. 4C), suggesting that as stimulation frequency increased, female mice refined the behavioral strategy by making more entries/exits to bypass the timeout period and maximize the amount of stimulation received.

This self-stimulation behavior is less robust in males. During acquisition trials, male mice learned to actively enter the active zone over the inactive zones during 30 Hz acquisition trial, but not during the 10-Hz or 20-Hz trials. There were no differences between the difference in the number of entries into the active zone versus into the inactive zones at any stimulation parameter (Fig. 3B). This suggests that although with higher frequency stimulation males learned to seek the active zone, activation of this pathway is not as salient to males as it is to females. This is especially apparent at 30 Hz, wherein the differences between the number of entries into the active versus inactive zones were significantly less in males as compared with females (Fig. 3A, B), as were the number of stimulation trains received (Fig. 4A, B). Though males spent more time in the active zone than in the inactive zones during both the 10-Hz and 30-Hz conditions, increasing the stimulation frequency did not significantly affect the difference between these values (Fig. 3D). That is, they were not spending significantly more time in the active zone at 30 Hz than they were at 10 Hz, suggesting that there may be a limit as to how reinforcing ILC-NAcSh stimulation is in males. Interestingly, while there were no sex differences in the difference between the number of entries into the active versus inactive zones at 20 Hz (Fig. 3A, B), there were significant differences in the number of stimulation trains received (Fig. 4A, B). This suggests that by 20 Hz stimulation, females had begun to refine the behavioral strategy to receive maximal stimulation, while males had not.

Despite the marked sex differences in the number of entries into the acquisition zone, there were no sex differences in distance or speed of travel. Starting with the baseline trial and continuing through each acquisition day trial, we found no sex differences in the average distance traveled or the average travel speed, indicating that optogenetic stimulation was not driving motor behavior differently in either sex. However, increasing stimulation frequency sequentially increased both distance traveled and travel speed. Together, these data are consistent with previous work suggesting that ILC stimulation is involved in purposeful motor pattern learning. Additionally, acquisition day testing established that ILC stimulation was rewarding, as mice sought the active zone over the inactive zones (Figs. 3, 4). These results are in line with data indicating that this pathway carries positive reward valence [10] and suggest that while the ILC-NAcSh circuit drives reward-seeking behavior through motor pattern activity in both sexes, this circuit is more sensitive to stimulation in females, supporting reports of higher motivation to seek psychostimulants and greater locomotive stereotypy in females [27–29, 67].

The second day of each test, the reversal day, allowed us to further parse out the behavioral strategies involved in reward-seeking behavior, including: (1) if the mice learned that the stimulation location was different, and (2) the strategy involved in seeking the stimulation in a new location, if it was learned at all. In females, increasing optogenetic frequency resulted in mice entering the active zone more than the inactive zones, but unlike acquisition trials, this was only significant at 20 and 30 Hz. The difference between the number of entries into the active zone versus into the inactive zones was blunted as compared with the acquisition day; significant differences were only found between 10 and 30 Hz (Fig. 6A). However, the number of stimulations received, and subsequently the amount of time stimulation was received, was significantly greater at both 20 Hz and 30 Hz as compared with 10 Hz, though there was no difference between the two higher stimulation parameters (Fig. 6A, C). Females showed significant differences between these two stimulation parameters in both the number of stimulations received (Fig. 7A) and the total time optogenetic stimulation was received (Fig. 7C), but not the total time spent in the active zone (Fig. 6C). Taken with acquisition day trials, these data suggest that female mice were able to learn the optimal motor activity patterns necessary to receive rewarding stimulation during the first day and apply and refine that behavior across time. Activation of this circuit may be part of the mechanism that drives females to be more vulnerable than males to relapse [68].

Males also learned to seek optogenetic stimulation in the reversal zone, spending significantly more time in the active than in the inactive zones at all frequencies (Fig. 6D), while only during the 10-Hz trial was there an increase the number of entries into the active zone over the inactive zones, there was a trend toward significance during the 30-Hz trial (Fig. 6B). There were no differences in the difference between entries into the active zone versus inactive zones or the difference between time spent in the active zone versus inactive zones at any stimulation frequency. There were no differences in the total number of stimulation trains received or the total time optogenetic stimulation was received at any parameter (Fig. 7B, D). However, during the reversal day sex differences notably decreased, suggesting that by the second day males were beginning to optimize the reward-seeking strategy in the same way females had.

Like acquisition trials, we found no sex differences in distance or travel speed during reversal trials, indicating that optogenetic stimulation was not driving motor behavior differently in either sex on the second day. Consistent with the acquisition trials, increasing stimulation frequency sequentially increased both the distance traveled and the travel speed (Fig. 8A–D). Within each sex there were no differences in either distance or travel speed between each respective acquisition and reversal day trial, indicating that optogenetic stimulation remained equally effective during day two. Finally, while we analyzed female mice at two different points in the estrous cycle, we found no differences between these phases. While cycle-dependent differences in behavior have been noted elsewhere [69], in the ILC-NAcSh circuit these were not apparent, thus the two groups of females were combined.

Whether or not we would see differences between phases if the full cycle had been assessed, or whether we would see differences between females and males, remains unclear. However, it has been demonstrated that sex differences in MSNs appear prepubertally, with female MSNs already displaying increased mEPSC frequency as compared with males. This effect is abolished with neonatal exposure to estradiol or testosterone, suggesting that many sex differences in MSN synaptic input and physiology are organizational in nature [70]. However, the activational effects of estradiol acting through an ERα mechanism on MSN physiology in the NAc as a whole have been shown [49–51]. Furthermore, given that glutamate concentrations in certain brain regions is influenced by the estrous cycle [33], parsing out estrous cycle differences may be an avenue of future studies. On the other hand, as the ILC-NAcSh circuit is only one portion of the larger motivation circuit, the effect of the estrous cycle may be less robust in this projection than in other circuits.

Along with glutamatergic signaling, estradiol is known to affect DAergic signaling in the VTA [71]. Not only do females display a higher proportion of DAergic neurons in the VTA [63], but these cells show sensitivity to the estrous cycle [71, 72]. During proestrus, when the circulating level of estradiol is high, basal firing rates are low. Conversely, during estrus, when the estradiol level has dropped, firing rates are at their highest [73, 74]. The interaction of glutamatergic projections from the ILC and DAergic projections from the VTA in the context of the estrous cycle (and indeed, between the sexes) and the subsequent effect on motivated behavior is an area ripe for further exploration.

To examine possible neurophysiological mechanisms underlying these sex differences in behavior, we assessed synaptic strength by recording optogenetically stimulated local field potentials, as well as performing whole-cell current clamp recordings to examine NAcSh MSN neuronal excitability. While optogenetic stimulation in vivo elicited more robust behavioral responses in females, ex vivo optogenetic stimulation elicited a greater response in males across all stimulation durations (Fig. 9B–D). This surprising result highlights a conundrum; in males, despite the presence of robust functional strength in the ILC-NAcSh pathway, activation of this pathway is weakly reinforcing, whereas in females the opposite is true.

On the other hand, when we directly assayed the intrinsic excitability of NAcSh MSNs, we found that females exhibited significantly greater excitability than males (Figs. 9F–I; 10A–D). Taken together with the behavioral data, one possible solution to this conundrum is that because MSNs in the female NAcSh exhibit heightened intrinsic excitability, they require less glutamatergic stimulation to elicit a maximal behavior response, whereas the reverse may be true in males. Interestingly, the data also suggest a potential scaling effect, wherein postsynaptic excitability is high in females, and therefore presynaptic glutamatergic inputs may balance this excitability by reducing the amount of neurotransmission to the MSNs. While this possible explanation has its merits, one caveat is that correlating a heterogenous population response such as our oLFP recordings with neuronal excitability measurements from individual neurons is not straightforward, and thus further studies would be needed to confirm this scaling hypothesis.

## Conclusions

Overall, the findings here indicate novel sex differences in the glutamatergic ILC-NAcSh circuit driving reward-seeking behavior. Our data further confirm that stimulation of the glutamatergic ILC-NAcSh circuit drives purposeful motor pattern behavior [18] and is particularly effective in females. Females were able to optimize their strategy during acquisition trials to obtain the greatest amount of stimulation, and the locomotive behaviors involved in seeking the reward increased sequentially with increased optogenetic stimulation frequency. They were also able to maintain this strategy during the reversal trials, indicating that motor patterns, rather than location, were salient. In comparison, males were less responsive. However, males showed greater responsivity to ex vivo optogenetic stimulation than did females at all stimulation durations, suggesting that glutamatergic synaptic strength is greater in males, and that higher stimulation may be needed for males to exhibit behavior comparable to females. Females exhibited greater intrinsic excitability in NAcSh MSNs, which may suggest that less glutamatergic input is necessary to elicit a robust MSN and subsequent behavioral response. Furthermore, as postsynaptic excitability is high, a reduction in presynaptic glutamate neurotransmission may balance the intrinsic excitability. While a succinct explanation of how the observed neurophysiological mechanisms account for sex differences in reward-seeking behavior remains unclear, future studies will aim to parse them out.

## Significance and perspectives

Here we present data demonstrating in mice fundamental sex differences in reward-seeking behavior, as well as inherent neurophysiological sex differences in the ILC-NAcSh circuit. Given the known differences in the motivation and reward system between the sexes, and documented sex differences in drug-seeking behavior in humans, parsing out the different neurophysiological mechanisms in the underlying circuitry is important to develop a comprehensive understanding of sex differences in substance use, and substance use disorders, with the ultimate goal of effective treatments.

### Supplementary Information

**Additional file 1: **There were no differences in any parameter between the groups of females in the two measured stages of the estrous cycle. Group 1 (light gray) underwent Acquisition trial days in proestrus and Reversal trial days in estrus, while Group 2 (dark gray) underwent Acquisition trial days in estrus and Reversal trial days in metestrus. There were no differences in the number of entries into the acquisition zone above the average of the number of entries into the inactive zones between females in group 1 (14.14 ± 11.46) and those in group 2 (19.75 ± 6.29) during the 10 Hz acquisition trial, as determined by a Student’s t-test (*p* = 0.66) (**A**). This was true during the 10 Hz reversal trial as well. Females in group 1 entered the active zone 20.43 ± 12.55 more times than the inactive zones and females in group 2 entered the active zone 26.25 ± 14.27 more times than the inactive zones (*p* = 0.78) (**B**). The total amount of time spent in the active zone above the average of the amount of time spent in the inactive zones did not differ between the two groups either. A Welch’s t-test indicated that during the 10 Hz acquisition trial, females in group 1 spent 32.29 ± 114.00 additional seconds in the active zone, and females in group 2 spent 86.24 ± 35.64 additional seconds (*p* = 0.66) (**C**). A Student’s t-test indicated that there were no differences during the 10 Hz reversal trial. Females in group 1 spent 344.20 ± 98.52 additional seconds in the active zone, while females in group 2 spent 213.30 ± 79.14 additional seconds (*p* = 0.32) (**D**). Additionally, Student’s t-tests indicated that there were no differences in distance traveled between the two groups during the 10 Hz acquisition trial (Group 1: 56.39 ± 16.48 m; Group 2: 82.54 ± 8.11 m; *p* = 0.15) (**E**) or the 20 Hz reversal trial (Group 1: 71.44 ± 17.28 m; Group 2: 98.44 ± 16.25 m; *p* = 0.28) (**F**). Finally, Student’s t-tests also indicated that there were no differences in speed during either the 10 Hz acquisition (Group 1: 0.03 ± 0.009 m/s; Group 2: 0.05 ± 0.005 m/s; *p* = 0.15) (**G**) or 10 Hz reversal trials (Group 1: 0.04 ± 0.01 m/s; Group 2: 0.05 ± 0.009 m/s; *p* = 0.28) (**H**).

**Additional file 2: **As in the 10 Hz trials, Group 1 (light gray) underwent Acquisition trial days in proestrus and Reversal trial days in estrus, while Group 2 (dark gray) underwent Acquisition trial days in estrus and Reversal trial days in metestrus. No differences were found during the 20 Hz acquisition trial in the number of entries into the acquisition zone above the average of the number of entries into the inactive zones between females in group 1 (88.14 ± 19.36) and those in group 2 (84.33 ± 16.79) as determined by a Student’s t-test (*p* = 0.89) (**A**). This was true during the 20 Hz reversal trial as well, wherein females in group 1 entered the active zone 85.14 ± 20.12 more times than the inactive zones and females in group 2 entered the active zone 51.83 ± 19.23 more times than the inactive zones (*p* = 0.26) (**B**). As with the number of entries, the total amount of time spent in the active zone above the average of the amount of time spent in the inactive zones did not differ between the two groups. There were no differences between the two groups of females in the amount of time spent in the active zone above that spent in the inactive zone, as determined by a Student’s t-test (Group 1: 698.80 ± 118.30 s; Group 2: 666.90 ± 130.70 s; *p* = 0.86) (**C**). This was true during the 20 Hz reversal trial as well, wherein females in group 1 spent 689.30 ± 156.60 additional seconds in the active zone, and females in group 2 spent 627.50 ± 162.20 additional seconds in the active zone. A Student’s t-test indicated no difference (*p* = 0.79) (**D**). Furthermore, Student’s t-tests indicated that there were no differences in distance traveled between the two groups during the 20 Hz acquisition trial (Group 1: 129.90 ± 7.10 m; Group 2: 141.40 ± 18.73 m; *p* = 0.54) (**E**) or the 20 Hz reversal trial (Group 1: 140.10 ± 7.08 m; Group 2: 129.00 ± 23.03 m; *p* = 0.61) (**F**). Finally, Student’s t-tests also indicated that there were no differences in speed during either the 20 Hz acquisition (Group 1: 0.07 ± 0.004 m/s; Group 2: 0.08 ± 0.01 m/s; *p* = 0.52) (**G**) or 20 Hz reversal trials (Group 1: 0.08 ± 0.004 m/s; Group 2: 0.07 ± 0.01 m/s; *p* = 0.60) (**H**).

**Additional file 3: ** No behavioral differences between phases of the estrous cycle during 30 Hz trials. Like the 10 Hz and 20 Hz trials, Group 1 (light gray) underwent Acquisition trial days in proestrus and Reversal trial days in estrus, while Group 2 (dark gray) underwent Acquisition trial days in estrus and Reversal trial days in metestrus. During the 30 Hz acquisition trial there were no differences in the number of entries into the acquisition zone above the average of the number of entries into the inactive zones between females in group 1 (138.60 ± 29.08) and those in group 2 (131.80 ± 23.21), as determined by a Student’s t-test (*p* = 0.88) (**A**). There were no differences during the 30 Hz reversal trial either. Females in group 1 entered the active zone 157.00 ± 39.31 more times than the inactive zones and females in group 2 entered the active zone 84.40 ± 26.35 more times than the inactive zones (*p* = 0.18) (**B**). During the 30 Hz acquisition trial there were no differences between the two groups of females in the amount of time spent in the active zone above that spent in the inactive zone, as determined by a Student’s t-test (Group 1: 788.70 ± 172.10 s; Group 2: 742.20 ± 114.30 s; *p* = 0.86) (**C**), nor were there differences between the two groups during the 30 Hz reversal trial (Group 1: 697.00 ± 214.40 s; Group 2: 696.60 ± 179.50 s; *p* = 0.99) (**D**). Finally, Student’s t-tests indicated that there were no differences in distance traveled between the two groups during the 30 Hz acquisition trial (Group 1: 177.20 ± 22.87 m; Group 2: 170.50 ± 23.25 m; *p* = 0.86) (**E**) or the 30 Hz reversal trial (Group 1: 172.10 ± 21.80 m; Group 2: 144.10 ± 16.09 m; *p* = 0.36) (**F**). Student’s t-tests further indicated that there were no differences in speed during either the 30 Hz acquisition (Group 1: 0.10 ± 0.01 m/s; Group 2: 0.09 ± 0.01 m/s; *p* = 0.86) (**G**) or 30 Hz reversal trials (Group 1: 0.10 ± 0.01 m/s; Group 2: 0.08 ± 0.009 m/s; *p* = 0.36) (**H**).

## Data Availability

The data that support the findings of this study are available from the corresponding author upon reasonable request.
