# Supplementary figures and images for: Sex differences in mouse infralimbic cortex projections to the nucleus accumbens shell
Source: Biol Sex Differ. 2023 Dec 11;14:87. doi: 10.1186/s13293-023-00570-3 (PMC10712109; doi:10.1186/s13293-023-00570-3)

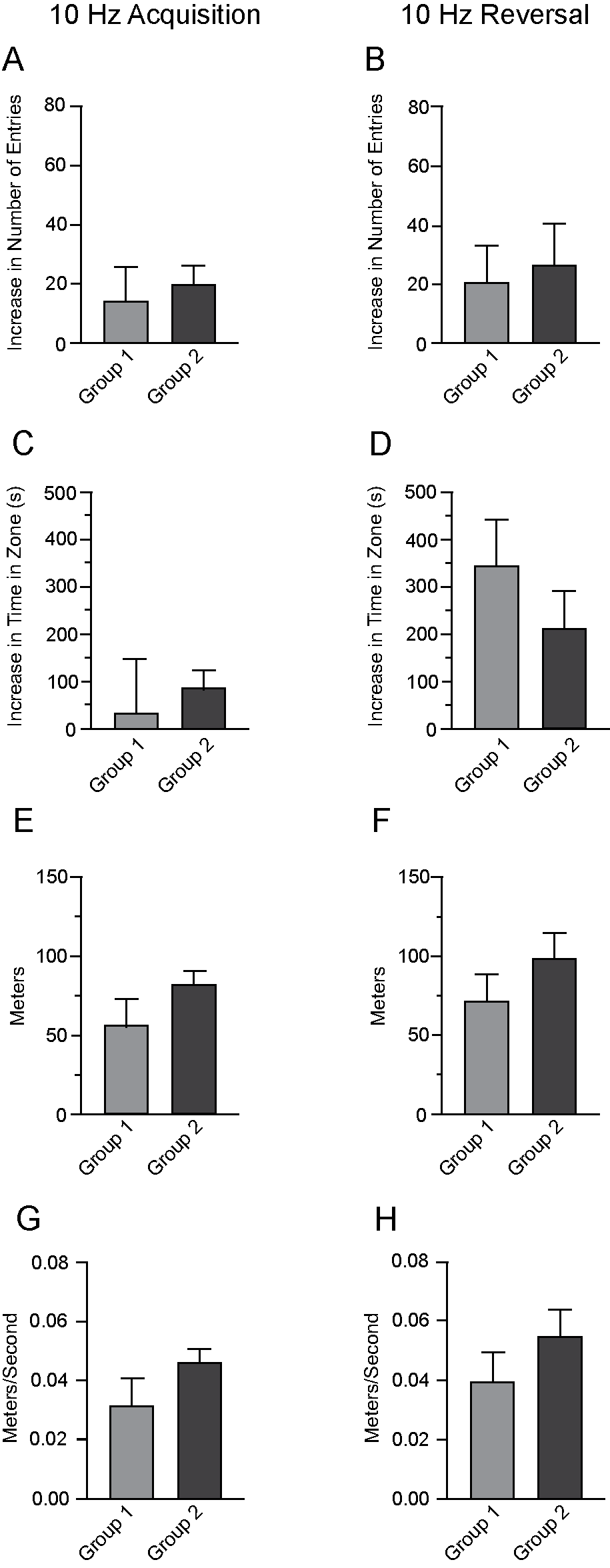

Supplement: Supplementary file 1 — Additional file 1: There were no differences in any parameter between the groups of females in the two measured stages of the estrous cycle. Group 1 (light gray) underwent Acquisition trial days in proestrus and Reversal trial days in estrus, while Group 2 (dark gray) underwent Acquisition trial days in estrus and Reversal trial days in metestrus. There were no differences in the number of entries into the acquisition zone above the average of the number of entries into the inactive zones between females in group 1 (14.14 ± 11.46) and those in group 2 (19.75 ± 6.29) during the 10 Hz acquisition trial, as determined by a Student’s t-test (p = 0.66) (A). This was true during the 10 Hz reversal trial as well. Females in group 1 entered the active zone 20.43 ± 12.55 more times than the inactive zones and females in group 2 entered the active zone 26.25 ± 14.27 more times than the inactive zones (p = 0.78) (B). The total amount of time spent in the active zone above the average of the amount of time spent in the inactive zones did not differ between the two groups either. A Welch’s t-test indicated that during the 10 Hz acquisition trial, females in group 1 spent 32.29 ± 114.00 additional seconds in the active zone, and females in group 2 spent 86.24 ± 35.64 additional seconds (p = 0.66) (C). A Student’s t-test indicated that there were no differences during the 10 Hz reversal trial. Females in group 1 spent 344.20 ± 98.52 additional seconds in the active zone, while females in group 2 spent 213.30 ± 79.14 additional seconds (p = 0.32) (D). Additionally, Student’s t-tests indicated that there were no differences in distance traveled between the two groups during the 10 Hz acquisition trial (Group 1: 56.39 ± 16.48 m; Group 2: 82.54 ± 8.11 m; p = 0.15) (E) or the 20 Hz reversal trial (Group 1: 71.44 ± 17.28 m; Group 2: 98.44 ± 16.25 m; p = 0.28) (F). Finally, Student’s t-tests also indicated that there were no differences in speed during either the 10 Hz acquisition (Group [file 13293_2023_570_MOESM1_ESM.tif]

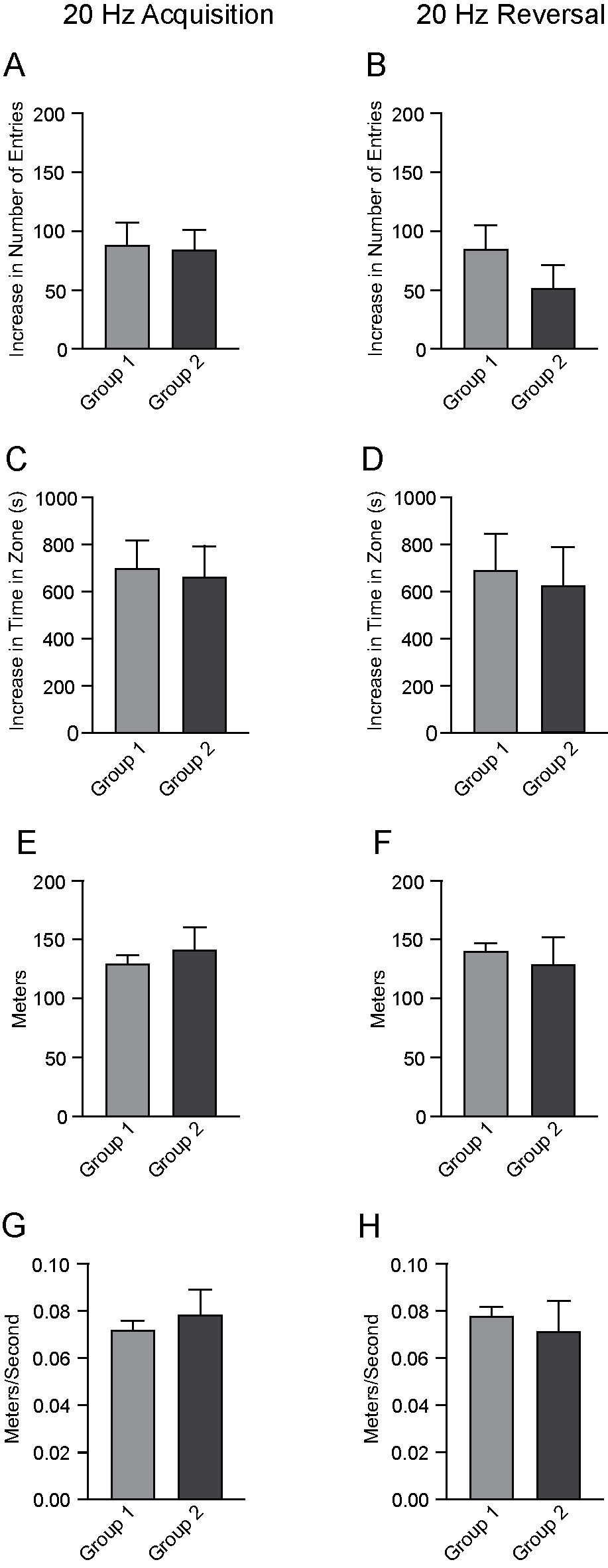

Supplement: Supplementary file 2 — Additional file 2: As in the 10 Hz trials, Group 1 (light gray) underwent Acquisition trial days in proestrus and Reversal trial days in estrus, while Group 2 (dark gray) underwent Acquisition trial days in estrus and Reversal trial days in metestrus. No differences were found during the 20 Hz acquisition trial in the number of entries into the acquisition zone above the average of the number of entries into the inactive zones between females in group 1 (88.14 ± 19.36) and those in group 2 (84.33 ± 16.79) as determined by a Student’s t-test (p = 0.89) (A). This was true during the 20 Hz reversal trial as well, wherein females in group 1 entered the active zone 85.14 ± 20.12 more times than the inactive zones and females in group 2 entered the active zone 51.83 ± 19.23 more times than the inactive zones (p = 0.26) (B). As with the number of entries, the total amount of time spent in the active zone above the average of the amount of time spent in the inactive zones did not differ between the two groups. There were no differences between the two groups of females in the amount of time spent in the active zone above that spent in the inactive zone, as determined by a Student’s t-test (Group 1: 698.80 ± 118.30 s; Group 2: 666.90 ± 130.70 s; p = 0.86) (C). This was true during the 20 Hz reversal trial as well, wherein females in group 1 spent 689.30 ± 156.60 additional seconds in the active zone, and females in group 2 spent 627.50 ± 162.20 additional seconds in the active zone. A Student’s t-test indicated no difference (p = 0.79) (D). Furthermore, Student’s t-tests indicated that there were no differences in distance traveled between the two groups during the 20 Hz acquisition trial (Group 1: 129.90 ± 7.10 m; Group 2: 141.40 ± 18.73 m; p = 0.54) (E) or the 20 Hz reversal trial (Group 1: 140.10 ± 7.08 m; Group 2: 129.00 ± 23.03 m; p = 0.61) (F). Finally, Student’s t-tests also indicated that there were no differences in speed during either the 20 Hz acquisition (Group 1 [file 13293_2023_570_MOESM2_ESM.tif]

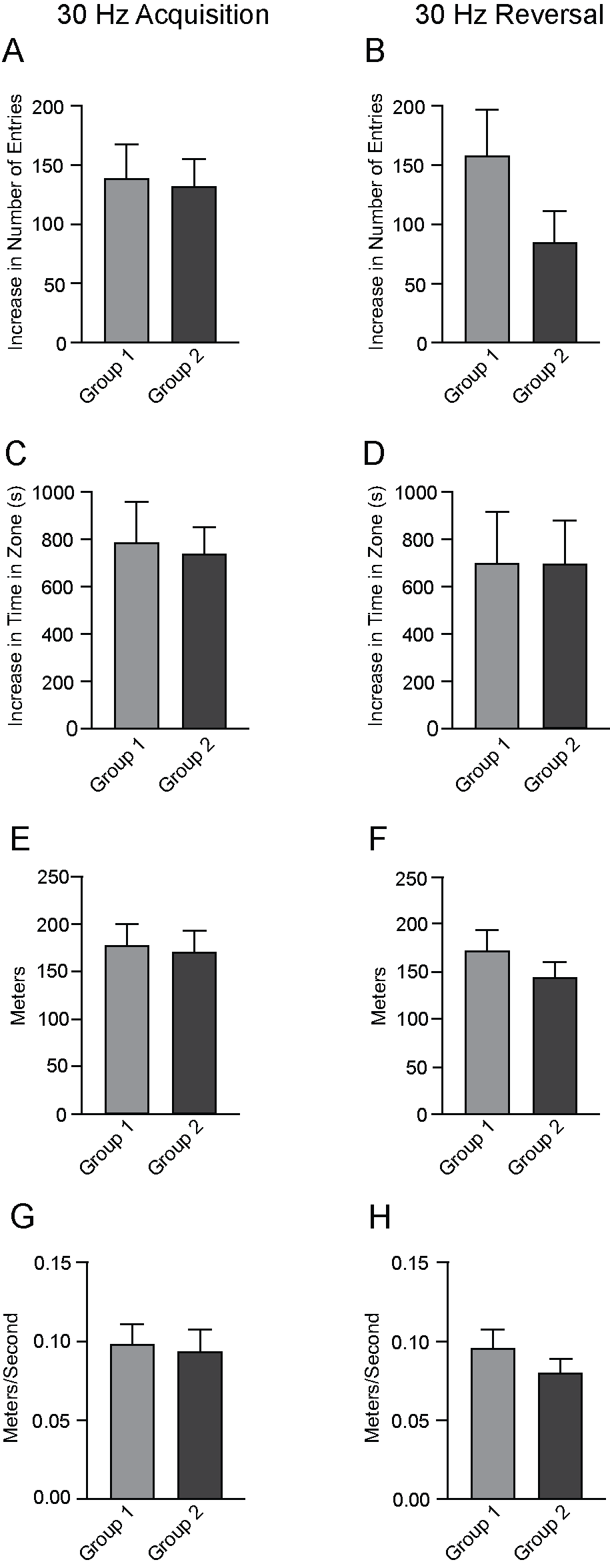

Supplement: Supplementary file 3 — Additional file 3: No behavioral differences between phases of the estrous cycle during 30 Hz trials. Like the 10 Hz and 20 Hz trials, Group 1 (light gray) underwent Acquisition trial days in proestrus and Reversal trial days in estrus, while Group 2 (dark gray) underwent Acquisition trial days in estrus and Reversal trial days in metestrus. During the 30 Hz acquisition trial there were no differences in the number of entries into the acquisition zone above the average of the number of entries into the inactive zones between females in group 1 (138.60 ± 29.08) and those in group 2 (131.80 ± 23.21), as determined by a Student’s t-test (p = 0.88) (A). There were no differences during the 30 Hz reversal trial either. Females in group 1 entered the active zone 157.00 ± 39.31 more times than the inactive zones and females in group 2 entered the active zone 84.40 ± 26.35 more times than the inactive zones (p = 0.18) (B). During the 30 Hz acquisition trial there were no differences between the two groups of females in the amount of time spent in the active zone above that spent in the inactive zone, as determined by a Student’s t-test (Group 1: 788.70 ± 172.10 s; Group 2: 742.20 ± 114.30 s; p = 0.86) (C), nor were there differences between the two groups during the 30 Hz reversal trial (Group 1: 697.00 ± 214.40 s; Group 2: 696.60 ± 179.50 s; p = 0.99) (D). Finally, Student’s t-tests indicated that there were no differences in distance traveled between the two groups during the 30 Hz acquisition trial (Group 1: 177.20 ± 22.87 m; Group 2: 170.50 ± 23.25 m; p = 0.86) (E) or the 30 Hz reversal trial (Group 1: 172.10 ± 21.80 m; Group 2: 144.10 ± 16.09 m; p = 0.36) (F). Student’s t-tests further indicated that there were no differences in speed during either the 30 Hz acquisition (Group 1: 0.10 ± 0.01 m/s; Group 2: 0.09 ± 0.01 m/s; p = 0.86) (G) or 30 Hz reversal trials (Group 1: 0.10 ± 0.01 m/s; Group 2: 0.08 ± 0.009 m/s; p = 0.36) (H). [file 13293_2023_570_MOESM3_ESM.tif]
